# Improving the Antioxidant Properties of *Calophyllum inophyllum* Seed Oil from French Polynesia: Development and Biological Applications of Resinous Ethanol-Soluble Extracts

**DOI:** 10.3390/antiox10020199

**Published:** 2021-01-30

**Authors:** Mathieu Cassien, Anne Mercier, Sophie Thétiot-Laurent, Marcel Culcasi, Emilie Ricquebourg, Alice Asteian, Gaëtan Herbette, Jean-Pierre Bianchini, Phila Raharivelomanana, Sylvia Pietri

**Affiliations:** 1 Aix Marseille Univ, CNRS, ICR , UMR 7273, SMBSO, 13397 Marseille , France; mathieu.cassien@univ-amu.fr (M.C.); anne.mercier@univ-amu.fr (A.M.); sophie.thetiot-laurent@univ-amu.fr (S.T.-L.); marcel.culcasi@univ-amu.fr (M.C.); emilie.ricquebourg@laposte.net (E.R.); alice.asteian@gmail.com (A.A.); 2 Univ Polynésie Française, IFREMER, ILM, IRD, EIO UMR 241, 98702 Faa’a Tahiti, French Polynesia; jbianchin1938@gmail.com (J.-P.B.); phila.raharivelomanana@upf.pf (P.R.); 3 Yelen Analytics, 13820 Ensuès–la-Redonne, France; 4 Aix Marseille Univ, CNRS, Spectropole, FSCM, 13397 Marseille, France; gaetan.herbette@univ-amu.fr

**Keywords:** *Calophyllum inophyllum* L., Tamanu oil, ethanol-soluble resin extracts, lipophilic antioxidants, phenolics, pyranocoumarins, antimicrobial, anti-inflammatory, UV protection, EPR spin trapping

## Abstract

Tamanu oil from *Calophyllum inophyllum* L. has long been used in traditional medicine. Ethanol extraction was found the best strategy for recovering bioactive compounds from the resin part of Tamanu oil, yielding two neutral and acidic resins fractions with high phenolics, flavonoids and pyranocoumarins concentrations. A further cascade of LPLC/HPLC separations of neutral and acidic resin fractions allowed identifying fifteen metabolites, and among them, calanolide D and 12-oxocalanolide A (both in neutral fraction) were first identified from a natural source. All these extracts, subfractions and isolated metabolites demonstrated increased free radical scavenging, antioxidant, anti-inflammatory, antimicrobial and antimycobacterial activity compared to Tamanu oil and its de-resinated lipid phase. Overall, these results could promote resinous ethanol-soluble Tamanu oil extracts as a useful multifaceted and renewable medicinal resource.

## 1. Introduction

*Calophyllum inophyllum* L. (Calophyllaceae) is an evergreen tree distributed in tropical areas of Africa, America, Asia and Pacific Islands. Many pharmacological properties from different parts of *C. inophyllum* such as leaves, bark and oil have been reported in traditional folk medicines, with variations in local uses [1]. Leaves are generally used as decoctions, to treat dermatoses or eye ailments, and the bark juice has purgative properties. Expressed from the seeds, *C. inophyllum* oil is widely used as a natural antiseptic and anti-inflammatory agent and for treating venous ulcers, rheumatisms and wounds [2]. Several contributions to the knowledge of the *mechanisms of action of* *C. inophyllum* oil have been reported, and its anti-inflammatory, anti-microbial and cytoprotective properties have been evidenced in cell models [3,4]. *C. inophyllum* oil is essentially composed of lipids (about 70–80%) [5], and of a resinous part gathering the main bioactive compounds (about 15–20%), such as sterols, coumarins, triterpenoids and flavonoids [1,2]. In recent decades, a series of pyranocoumarins in seeds or leaves of *Calophyllum* genus plants have been identified, belonging to the calanolide, inophyllum or cordatolide series [6,7,8,9]; e.g., calanolides A, B and pseudocalanolides C and D are found in *C. lanigerum* [6]. In the *C. inophyllum* oil found worldwide, several 4-phenylcoumarin derivatives were isolated, such as callophylolide and inophyllolides, including inophyllums B, C, E and P [7,8,10,11]. Remarkable biochemical and pharmacological properties have been reported for some of these constituents; e.g., (+)-calanolide A is a non-nucleosidic reverse transcriptase inhibitor [9] exhibiting protection against *Mycobacterium tuberculosis* [12]. Callophylolide and inophyllums C and E were found to exhibit antimicrobial and cytotoxic activities [11] and preventive action against cancer induction by the Epstein-Barr virus [13]. Calophyllic and isocalophyllic acids, found in the *C. inophyllum* leaves, demonstrated antidyslipidemic and weak antioxidant activities [14].

In French Polynesia, *C. inophyllum* is locally called *Tamanu* and has a great heritage interest as a sacred tree. Tamanu oil (TO), the non-edible cold-pressed oil from dried nuts is widely used in traditional medicine and as a component for cosmetics commercialized in French Polynesia or for exportation. Recently, remarkable wound healing and antimicrobial properties of crude *C. inophyllum* oils from several origins have been reported, with variations depending on their variable compositions [15]. Other data revealed a strong enhancement of metabolic processes involved in cell adhesion and proliferation in keratinocytes upon treatment with TO emulsified with olive oil [16].

However, despite its great potential interest, major drawbacks of using TO as a raw material for therapeutic and cosmetic applications arise both from a very high lipophilicity and the weak amount of potentially bioactive compounds contained in the minor soluble phase [17]. This latter issue could be solved by ethanol extraction, a process that would allow recovering the largest amount of TO metabolites. To illustrate, three new pyranocoumarins with an unprecedented C-4 substituent, namely, tamanolide and tamanolides D and P [18], and two other new neoflavonoids, i.e., tamanolides E1 and E2 [19], were recently characterized from this ethanolic resin fraction. In line with the above, ethanolic extracts of *C. inophyllum* seed exhibited antiarthritic activities in rats, suggesting that a significant amount of phytoconstituents are bioavailable [20].

In our continuous interest in developing easy handling pharmaceutical preparations from natural sources of great economic potential [21,22], the purpose of the present study was (i) to prepare novel soluble TO extracts and subfractions with enhanced inhibition properties toward a host of reactive oxygen species (ROS); (ii) to screen them for their antioxidant, anti-inflammatory and antimicrobial properties using a battery of physicochemical and biochemical tests; and (iii) to further isolate and characterize in these TO fractions those compounds showing the greatest potential for biological applications.

## 2. Materials and Methods

### 2.1. Extraction and Fractionation of Tamanu Oil and Resins

#### 2.1.1. Chemicals and Instrumentation

Folin-Ciocalteu reagent, gallic acid, rutin and all other chemicals and solvents were of analytical grade from Acros Organics-Fisher Scientific (Illkirch, France) or Sigma-Aldrich (Saint-Quentin Fallavier, France). Deuterated solvents were purchased from Euriso-Top (Gif-sur-Yvette, France), and TLC silica gel plates were purchased from Merck (Darmstadt, Germany). ^1^H NMR spectra were recorded in CDCl_3_ on a Bruker Avance DRX 500 spectrometer operating at 500 MHz. Chemical shifts (δ) are reported in ppm relative to internal tetramethylsilane (TMS). Coupling constants are expressed in Hertz (Hz). Splitting patterns are as follows: s = singlet; d = doublet; dd = doublet of doublets; t = triplet; q = quadruplet; dq = doublet of quadruplets; tq = triplet of quadruplets; sext = sextet; m = multiplet; b = broad peak. Optical rotations were measured using a SCP 200 polarimeter (Anton Paar; Courtaboeuf, France) with a 0.2-cm path length. Low-Pressure Liquid Chromatography (LPLC) was performed on a glass chromatography column (600 mm × 50 mm, 1 L) filled with silica gel 60 (SiO_2_, Prolabo, Fontenay-sous-Bois, France), connected to a low-pressure pump operating at a flow-rate of 6 mL/min. Preparative HPLC was performed on an Agilent HP 1100 Series system (Massy, France) equipped with a quaternary pump (G 1311A, Agilent), a standard autosampler (ALS G1313A, Agilent), silica columns (250 × 10 mm i.d., 10 µm, LiChrospher 100 DIOL, Merck, Darmstadt, Germany), at a flow-rate of 10 mL/min and monitored by a UV-DAD diode-array detector (G1315A, Agilent) using a preparative cell (3 mm path) at a defined detection wavelength. Spectrophotometric measurements were performed on an Infinite M200 microplate reader (TECAN infinite M200, Switzerland). Mass spectrometry (MS) analyses were carried out using a 3200 QTRAP mass spectrometer (Applied Biosystems SCIEX).

#### 2.1.2. Extraction of Ethanol-Soluble Resins from Tamanu Oil, Separation of Neutral and Acidic Fractions and Determination of Total Phenolic Content (TPC) and Total Flavonoid Content (TFC)

**Scheme 1:**
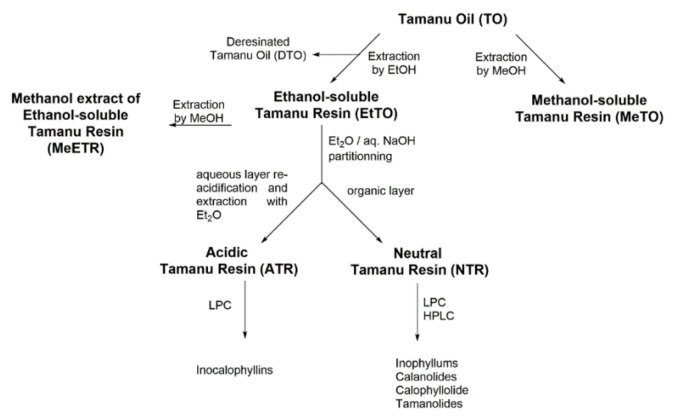
Extraction and fractionation process of Tamanu oil (TO) into methanol soluble (MeTO) and ethanol-soluble (EtTO) resins and subsequent neutral (NTR), acidic (ATR) and methanolic (MeETR) fractions preparation.

The whole process is shown in Scheme 1. Mature seeds of *C. inophyllum* were collected on the seashore of Huahine Island in French Polynesia, from September to October 2014, and manually crushed. The almonds were sun-dried for two weeks, and TO was obtained by cold pressing, followed by filtration to remove residual water. The recovered oil (1800 g) was first extracted with 70 mL of 96% *v*/*v* ethanol to remove a gummy dark green solid. The filtrate was then extracted with ethanol (3 × 1 L), and the solvent was removed under vacuum to afford the ethanol-soluble extract (EtTO, 285 g) as a brown resin. Residual de-resinated Tamanu oil (DTO) was kept for further experiments. Afterwards, EtTO (238 g) was dissolved in diethyl ether (2 L) and extracted with aqueous NaOH (10% *v*/*v*) until pH 10. The resulting organic layer was washed with distilled water until neutrality, dried over anhydrous MgSO_4_, filtered and concentrated to give a neutral Tamanu resin fraction (NTR, 119 g). The aqueous layer was carefully acidified until pH 4 with H_2_SO_4_ (1 M) under cooling, extracted with diethyl ether (3 × 100 mL), dried over anhydrous MgSO_4_, filtered and concentrated to give the acidic Tamanu resin fraction (ATR, 115 g).

TPC and TFC of TO and extracts were assessed using the Folin-Ciocalteu reagent and aluminium chloride complexation, respectively [23]. TPC was evaluated using a gallic acid calibration curve in methanol and expressed as milligrams of gallic acid equivalents (GAE) per gram of extract weight. TFC was evaluated using a rutin calibration curve in ethanol and expressed as milligrams of rutin equivalents (RUE) per gram of extract weight.

#### 2.1.3. Preparation of TO and EtTO Methanol Soluble Extracts

TO or EtTO were dissolved in methanol (1 g/mL) under agitation for 2 h at 22 °C, and vortexed for 4 min. The upper layers were collected, and the extraction process was performed two more times. Combined extracts from TO and EtTO were centrifuged (3000 rpm, 10 min, 4 °C) and the supernatants were evaporated until dryness to afford the methanol soluble Tamanu oil (MeTO; 18.2% *w*/*w* yield) and methanol soluble resin (MeETR, 25.3% *w*/*w* yield) extracts, respectively. TPC and TFC contents were determined for each extract as described above.

#### 2.1.4. LPLC Analysis of NTR and ATR Fractions

NTR (10 g) in 50 mL dichloromethane was mixed with 50 g silica gel 60. After solvent removal at room temperature, the impregnated silica was poured onto a glass column pre-filled with silica gel in *n*-hexane. LPLC was performed starting with *n*-hexane followed by the following step-gradient: *n*-hexane/AcOEt (10/90, 15/85, 20/80, 30/70, 40/60 and 50/50 *v*/*v*, 1 L each). Effluent fractions were monitored by thin-layer chromatography (TLC) and gathering similar fractions yielded a total of ten NTR subfractions termed as NTR I–NTR X. The whole process was repeated to allow a total amount of 70 g of NTR to be treated. A similar procedure was applied to ATR (6 × 10 g) using the step-gradient: *n*-hexane/AcOEt (10/90, 20/80, 40/60, and 80/20 *v*/*v*, 1 L each). From TLC monitoring, a total of four ATR subfractions termed as ATR I–ATR IV were then obtained.

#### 2.1.5. HPLC Separation of NTR and ATR Subfractions

HPLC separation and subfractions preparation was achieved according to previous procedures [18]. Six subfractions (50 mg) with moderate polarity and giving a blue spot with the vanillin/H_2_SO_4_ TLC stain (fractions NTR IV-VII and ATR II, III; TLC: 0.3 < R_f_ < 0.6, *n*-hexane-AcOEt, 7/3, *v*/*v*) were dissolved in 5 mL of a mixture of *n*-hexane/isopropanol (3/97, *v*/*v*). After filtration, the residues were fractionated by preparative HPLC using *n*-hexane/isopropanol (3/97, *v*/*v*) in isocratic conditions at a flow rate of 10 mL/min, with UV/Vis monitoring at 300 nm. The major compounds were manually collected and concentrated under vacuum. Fifteen phytoconstituents (pure compounds or mixtures) were then identified by comparing their NMR (^1^H and ^13^C), MS and absorption spectra with literature data. The recovered compounds are given as follows (LPLC fraction/HPLC peak, mg): inophyllum P (NTR IV/2, 20.7 mg); tamanolide P (NTR IV/3, 56.4 mg in mixture with inophyllum P and calanolide B, this fraction was termed as Fraction P); calophyllolide (NTR V/3, 26.2 mg); calanolide GUT 70 (NTR V/5, 32.9 mg); inophyllum C (NTR VI/1, 38.8 mg and NTR VII/5, 14.9 mg); 12-oxocalanolide A (NTR VI/2, 7.0 mg); inophyllum E (NTR VI/3, 19.4 mg); tamanolide D (NTR VI/4, 26.3 mg and inophyllum D and epi-calanolide C as a mixture, termed as Fraction D); tamanolide (NTR V/4, 28.6 mg); calanolide D (NTR VII/1, 10.0 mg); calanolide A (NTR VII/2, 7.0 mg); and inocalophyllin B (ATR II/1, 40.1 mg). Further separation on fraction NTR VI/4, driven as reported [18,24], yielded tamanolide D (1.1 mg) with a HPLC purity > 95%. Analytical HPLC controls were carried out on three different systems using either silica (*n*-hexane/isopropanol, 93/7, *v*/*v* or *n*-hexane/AcOEt, 78/22, *v*/*v*) or reversed-phase (C_18_) (acetonitrile/water/TFA, 85/15/0.1 *v*/*v*/*v*) columns.

These eleven metabolites showed HPLC purity > 95%, and their ^1^H- (see below), ^13^C-NMR and MS spectra are shown in Supplementary Information.

The composition of Fraction P was determined by HPLC as follows: inophyllum P (70%), tamanolide P (20%), and calanolide B (10%). Similarly the composition of Fraction D was determined as follows: tamanolide D (40%), inophyllum D (40%), and epi-calanolide C (20%). For both mixtures, ^1^H-NMR spectra are shown in Supplementary Information.

Nine of the isolated derivatives and Fraction P were obtained in reasonable amounts for further use in antioxidant and biological studies.

#### 2.1.6. UV, ^1^H-NMR and Optical Characteristics of Isolated TO Metabolites

*Inocalophyllin B.* UV (*n*-hexane/AcOEt, 78/22, *v*/*v*): λ_max_ (nm) 312, 282. ^1^H-NMR (CDCl_3_): δ 0.86 (t, *J* = 7.3 Hz, 3H, H-15), 1.14 (d, *J* = 7.0 Hz, 3H, H-22), 1.20 (m, *J* = 7.0 Hz, 2H, H-14), 1.53 (d, *J* = 7.0 Hz, 3H, H-21), 1.53 (bs, 3H, H-29), 1.54 (bs, 3H, H-19), 1.56 (bs, 3H, H-20), 1.58 (bs, 2H, H-32), 1.64 (bs, 3H, H-28), 1.76-1.82 (m, 2H, H-13), 1.87 (dd, *J* = 10.5 and 7.6 Hz, 1H, H-25), 1.95 (m, 1H, H-24), 1.99 (d, *J* = 12.7 Hz, 1H, H-23), 2.12 (dd, *J* = 12.7 and 1.0 Hz, 1H, H-23), 2.45 (bdd, *J* = 13.5 and 8.5 Hz, 1H, H-8), 2.48 (dq, *J* = 13.5 and 6.6 Hz, 1H, H-11), 2.61 (bdd, *J* = 13.5 and 6.8 Hz, 1H, H-8), 2.69 (dd, *J* = 15.6 and 6.4 Hz, 1H, H-3), 2.85 (dd, *J* = 15.6 and 8.3 Hz, 1H, H-3), 3.47 (m, 1H, H-4), 4.08 (dq, *J* = 13.5 and 6.4 Hz, 1H, H-10), 4.53 (dq, *J* = 10.3 and 1.4 Hz, 1H, H-31), 4.56 (dq, *J* = 12.3 and 1.4 Hz, 1H, H-31), 4.70 (tq, *J* = 6.9 and 1.4 Hz, 1H, H-7), 4.89 (m, *J* = 6.8 and 1.4 Hz, 1H, H-26), 12.32 (s, 1H, 12b-OH). Spectroscopic data (Figures S1 and S14) were in good agreement with the literature [25].

*Inophyllum C*. UV (*n*-hexane/AcOEt, 78/22, *v*/*v*): λ_max_ (nm) 300, 268, 258. ^1^H-NMR (CDCl_3_): δ 0.95 (s, 3H, H-20), 0.96 (s, 3H, H-19), 1.26 (d, *J* = 6.9 Hz, 3H, H-22), 1.56 (d, *J* = 6.2 Hz, 3H, H-21), 2.60 (dq, *J* = 11.1 and 6.9 Hz, 1H, H-11), 4.33 (dq, *J* = 11.1 and 6.3 Hz, 1H, H-10), 5.43 (d, *J* = 10.1 Hz, 1H, H-7), 6.07 (s, 1H, H-3), 6.56 (d, *J* = 10.1 Hz, 1H, H-8), 7.40 (m, 5H, H_phenyl_). Spectroscopic data (Figure S2) were in good agreement with the literature [26].

*Inophyllum D*. ^1^H-NMR (CDCl_3_): δ 0.79 (d, *J* = 7.3 Hz, 3H, H-22), 0.93 (s, 6H, H-19 and H-20), 1.42 (d, *J* = 6.5 Hz, 3H, H-21), 2.03 (qt, *J* = 7.2 and 2.1 Hz, 1H, H-11), 4.54 (qd, *J* = 6.6 and 2.2 Hz, 1H, H-10), 4.92 (d, *J* = 2.2 Hz, 1H, H-12), 5.35 (d, *J* = 10.0 Hz, 1H, H-7), 5.96 (s, 1H, H-3), 6.55 (d, *J* = 10.1 Hz, 1H, H-8), 7.23 (m, 2H, H_phenyl_), 7,35 (m, 3H, H_phenyl_). Spectroscopic data (Figure S12) were in good agreement with the literature [10].

*Inophyllum E*. UV (*n*-hexane/AcOEt, 78/22, *v*/*v*): λ_max_ (nm) 300, 268, 258. ^1^H-NMR (CDCl_3_): δ 0.97 (s, 3H, H-20), 0.99 (s, 3H, H-19), 1.20 (d, *J* = 7.2 Hz, 3H, H-22), 1.44 (d, *J* = 6.6 Hz, 3H, H-21), 2.73 (dq, *J* = 7.2 and 3.5 Hz, 1H, H-11), 4.74 (dq, *J* = 6.5 and 3.4 Hz, 1H, H-10), 5.43 (d, *J* = 10.1 Hz, 1H, H-7), 6.07 (s, 1H, H-3), 6.57 (d, *J* = 10.1 Hz, 1H, H-8), 7.32 (m, 5H, H_phenyl_). Spectroscopic data (Figure S3) were in good agreement with the literature [26].

*Inophyllum P*. UV (*n*-hexane/AcOEt, 78/22, *v*/*v*): λ_max_ (nm) 335, 288, 278. [α]^D^_25_ = +19.8° (c 0.8, CHCl_3_). ^1^H-NMR (CDCl_3_): δ 0.95 (s, 3H, H-19), 0.95 (s, 3H, H-20), 1.18 (d, *J* = 7.0 Hz, 3H, H-22), 1.45 (d, *J* = 6.3 Hz, 3H, H-21), 1.78 (ddq, *J* = 10.5, 3.4 and 7.0 Hz, 1H, H-11), 4.30 (dq, *J* = 10.7 and 6.3 Hz, 1H, H-10), 5.38 (d, *J* = 10.0 Hz, 1H, H-7), 5.05 (d, *J* = 3.3 Hz, 1H, H-12), 5.98 (s, 1H, H-3), 6.55 (d, *J* = 10.0 Hz, 1H, H-8), 7.3 (m, 5H, H_phenyl_). Spectroscopic data (Figure S4) were in good agreement with the literature [26].

*Calophyllolide*. UV (*n*-hexane/AcOEt, 78/22, *v*/*v*): λ_max_ (nm) 292, 270. ^1^H-NMR (CDCl_3_): δ 0.98 (s, 6H, H-19 and H-20), 1.91 (d, *J* = 7.0 Hz, 3H, H-21), 2.02 (s, 3H, H-22), 3.77 (s, 3H, H-23), 5.49 (d, *J* = 10.0 Hz, 1H, H-7), 6.03 (s, 1H, H-3), 6.47 (d, *J* = 10.0 Hz, 1H, H-8), 6.58 (bq, *J* = 7.0, 1H, H-10), 7.33 (m, 5H, H_phenyl_). Spectroscopic data (Figure S5) were in good agreement with the literature [26].

*12-Oxocalanolide A*. UV (*n*-hexane/AcOEt, 78/22, *v*/*v*): λ_max_ (nm) 294, 266, 258. ^1^H-NMR (CDCl_3_): δ 1.02 (t, *J* = 7.5 Hz, 3H, H-15), 1.21 (d, *J* = 6.9 Hz, 1H, H-19), 1.52 (s, 3H, H-17), 1.54 (d, *J* = 6.3 Hz, 3H, H-18), 1.55 (s, 3H, H-16), 1.64 (sext, *J* = 7.8 Hz, 2H, H-14), 2.55 (dq, *J* = 11.1 and 6.9 Hz, 1H, H-11), 2.88 (m, 2H, H-13), 4.29 (dq, *J* = 11.1 and 6.3 Hz, 1H, H-10), 5.59 (d, *J* = 10.0 Hz, 1H, H-7), 6.04 (bs, 1H, H-3), 6.65 (d, *J* = 10.0 Hz, 1H, H-8). Spectroscopic data (Figure S6) were in good agreement with the literature [27].

*Epi-calanolide C.*^1^H-NMR (CDCl_3_): δ (d, *J* = 7.1 Hz, 3H, H-22), 1.03 (t, *J* = 7.3 Hz, 3H, H-15), 1.42 (d, *J* = 6.5 Hz, 3H, H-21), 1.48 (s, 6H, H-19 and H-20), 1.65 (m, 2H, H-14), 2.03 (qt, *J* = 7.2 and 2.1 Hz, 1H, H-11), 2.89 (m, 2H, H-13), 4.54 (qd, *J* = 6.6 and 2.2 Hz, 1H, H-10), 4.86 (d, *J* = 2.1 Hz, 1H, H-12), 5.53 (d, *J* = 10.0 Hz, 1H, H-7), 5.95 (t, *J* = 0.8 Hz, 1H, H-3), 6.65 (d, *J* = 10.0 Hz, 1H, H-8). Spectroscopic data (Figure S12) were in good agreement with the literature [28].

*Calanolide A*. UV (*n*-hexane/AcOEt, 78/22, *v*/*v*): λ_max_ (nm) 258, 266. ^1^H-NMR (CDCl_3_): δ 1.03 (t, *J* = 7.3 Hz, 3H, H-15), 1.15 (d, *J* = 6.8 Hz, 3H, H-19), 1.46 (d, *J* = 6.4 Hz, 3H, H-18), 1.46 (s, 3H, H-17), 1.51 (s, 3H, H-16), 1.66 (m, 2H, H-14), 1.93 (ddq, *J* = 9.0, 7.8 and 6.8 Hz, 1H, H-11), 2.89 (m, 2H, H-13), 3.60 (bs, 1H, -OH), 3.92 (dq, *J* = 9.0 and 6.4 Hz, 1H, H-10), 4.74 (d, *J* = 7.8 Hz, 1H, H-12), 5.54 (d, *J* = 10.0 Hz, 1H, H-7), 5.94 (bs, 1H, H-3), 6.62 (d, *J* = 10.0 Hz, 1H, H-8). Spectroscopic data (Figures S7 and S15) were in good agreement with the literature [26].

*Calanolide B*. ^1^H-NMR (CDCl_3_): δ 1.02 (t, *J* = 7.3 Hz, 3H, H-15), 1.15 (d, *J* = 7.1 Hz, 3H, H-19), 1.48 (d, *J* = 6.4 Hz, 3H, H-18), 1.48 (s, 6H, H-16 and H-17), 1.64 (sext, *J* = 7.3 Hz, 2H, H-14), 1.73 (ddq, *J* = 10.5, 6.4 and 2.8 Hz, 1H, H-11), 2.78 (bs, 1H, -OH), 2.87 (m, 2H, H-13), 4.25 (dq, *J* = 10.5 and 6.4 Hz, 1H, H-10), 4.96 (d, *J* = 2.8 Hz, 1H, H-12), 5.52 (d, *J* = 9.9 Hz, 1H, H-7), 5.93 (bs, 1H, H-3), 6.63 (d, *J* = 9.9 Hz, 1H, H-8). Spectroscopic data (Figure S13) were in good agreement with the literature [26].

*Calanolide D*. UV (*n*-hexane/AcOEt, 78/22, *v*/*v*): λ_max_ (nm) 294, 266, 258. ^1^H-NMR (CDCl_3_): δ 1.02 (t, *J* = 7.3 Hz, 3H, H-15), 1.15 (d, *J* = 7.2 Hz, 3H, H-19), 1.41 (d, *J* = 6.6 Hz, 3H, H-18), 1.53 (s, 3H, H-17), 1.54 (s, 3H, H-16), 1.64 (m, 2H, H-14), 2.68 (dq, *J* = 3.4 and 7.2 Hz, 1H, H-11), 2.88 (m, 2H, H-13), 4.69 (dq, *J* = 3.4 and 6.6 Hz, 1H, H-10), 5.59 (d, *J* = 10.1 Hz, 1H, H-7), 6.04 (bs, 1H, H-3), 6.65 (d, *J* = 10.0 Hz, 1H, H-8). Spectroscopic data (Figure S8) were in good agreement with the literature [29].

*Calanolide GUT 70*. UV (*n*-hexane/AcOEt, 78/22, *v*/*v*): λ_max_ (nm) 292, 272. ^1^H-NMR (CDCl_3_): δ 1.05 (t, *J* = 7.3 Hz, 3H, H-15), 1.53 (s, H-16, 6H, H-17), 1.60 (m, 2H, H-14), 1.86 (d, *J* = 7.0 Hz, H3H,-18), 1.98 (s, 3H, H-19), 2.81 (t, *J* = 7.6 Hz, 2H, H-13), 3.76 (s, 3H, H-20), 5.66 (d, *J* = 10.0 Hz, 1H, H-7), 6.01 (s, 1H, H-3), 6.49 (q, *J* = 6.9 Hz, 1H, H-10), 6.55 (d, *J* = 10.0 Hz, 1H, H-8). Spectroscopic data (Figures S9 and S16) were in good agreement with the literature [26].

*Tamanolide*. UV (*n*-hexane/AcOEt, 78/22, *v*/*v*): λ_max_ (nm) 292, 266. [α]^D^_25_ = −3.3° (c 0.005, CHCl_3_). ^1^H-NMR (CDCl_3_): δ 0.98 (t, *J* = 6.7 Hz, 3H, H-15), 1.23 (d, *J* = 6.5 Hz, 3H, H-16), 1.43 (m, 1H, H-14a), 1.52 (s, 3H, H-19), 1.52 (s, 3H, H-20), 1.78 (m, 1H, H-14b), 1.86 (dq, *J* = 6.9 and 0.8 Hz, 3H, H-21), 1.97 (bs, 3H, H-22), 3.73 (s, *J* = 6.5 Hz, 3H, OCH_3_), 3.83 (sext, *J* = 6.5 Hz, 1H, H-13), 5.66 (d, *J* = 10.0 Hz, 1H, H-7), 6.11 (s, 1H, H-3), 6.49 (qq, *J* = 6.9 and 1.1 Hz, 1H, H-10), 6.55 (d, *J* = 10.0 Hz, 1H, H-8). Spectroscopic data (Figure S10) were in good agreement with the literature [18].

*Tamanolide D*. UV (*n*-hexane/AcOEt, 78/22, *v*/*v*): λ_max_ (nm) 296, 248. [α]^D^_25_ = −40.5° (c 0.006, CHCl_3_). ^1^H-NMR (CDCl_3_): δ 0.78 (d, *J* = 7.2 Hz, 3H, H-22), 0.95 (t, *J* = 7.3 Hz, 3H, H-15), 1.20 (d, *J* = 6.9 Hz, 3H, H-16), 1.41 (d, *J* = 6.5 Hz, 3H, H-21), 1.44 (m, 1H, H-14a), 1.46 (s, 3H, H-20), 1.47 (s, 3H, H-19), 1.76 (m, 1H, H-14b), 2.01 (qt, *J* = 7.2 and 2.0 Hz, 1H, H-11), 3.84 (sext, *J* = 6.6 Hz, 1H, H-13), 4.49 (qd, *J* = 6.5 and 2.0 Hz, 1H, H-10), 4.83 (d, *J* = 2.0 Hz, 1H, H-12), 5.52 (d, *J* = 10.0 Hz, 1H, H-7), 6.06 (s, 1H, H-3); 6.64 (d, *J* = 10.0 Hz, 1H, H-8). Spectroscopic data (Figures S11 and S17) were in good agreement with the literature [18].

*Tamanolide P*. ^1^H-NMR (CDCl_3_): δ 0.99 (t, *J* = 7.0 Hz, 3H, H-15), 1.18 (d, *J* = 7.0 Hz, 3H, H-22), 1.23 (d, *J* = 6.7 Hz, 3H, H-16), 1.45 (d, *J* = 6.3 Hz, 3H, H-21), 1.45 (m, 1H, H-14a), 1.50 (s, 3H, H-20), 1.50 (s, 3H, H-19), 1.79 (m, 1H, H-14b), 1.79 (dqd, *J* = 10.8, 7.0 and 3.2 Hz, 1H, H-11), 3.88 (sext, *J* = 6.7 Hz, 1H, H-13), 4.29 (qd, *J* = 10.8 and 6.3 Hz, 1H, H-10), 4.99 (d, *J* = 3.2 Hz, 1H, H-12), 5.55 (d, *J* = 10.0 Hz, 1H, H-7), 6.08 (s, 1H, H-3), 6.66 (d, *J* = 10.0 Hz, 1H, H-8). Spectroscopic data (Figure S13) were in good agreement with the literature [18].

### 2.2. Antioxidant Assays

#### 2.2.1. Chemicals, Enzymes and Instrumentation

The standards 6-hydroxy-2,5,7,8-tetramethylchroman-2-carboxylic acid (Trolox) and 2,2′-azobis(2-amidinopropane) dihydrochloride (AAPH) were from Acros Organics. Glycine buffer, Tris-HCl buffer, xanthine, xanthine oxidase (XO; from buttermilk), proteinase K (PTA), bovine serum albumin (BSA), diclofenac sodium salt, 1,1′-diphenyl-2-picrylhydrazyl (DPPH), lucigenin (*N*,*N*-dimethyl-9,9-biacridinium dinitrate), 2-thiobarbituric acid (TBA), allopurinol, luminol (5-amino-2,3-dihydro-phthalazine-1,4-dione), *tert*-butylhydroperoxide (*t*BuOOH), superoxide dismutase (SOD), catalase (CAT) and all other reagents were from Sigma-Aldrich. Phosphate-buffered saline (PBS) was from Life Technology Corp. (St Aubin, France).

DPPH decay and enzyme inhibition assays were performed on microplates. Luminescence was measured at room temperature on a Sirius luminometer (Titertek Berthold, Pforzheim, Germany) operating at 300–600 nm.

#### 2.2.2. Xanthine-Oxidase Inhibition

All reactants were dissolved in PBS (0.2 M, pH 7.5). The experiments were performed at 37 °C in 96-wells microplates with a final volume of 250 µL/well [30]. Wells were filled with 150 µL of either the test extracts (15–260 µg/mL), allopurinol (0.25–5 µM) or PBS (blank controls). To all wells, 25 µL of EDTA (0.1 mM in PBS) and 25 µL of xanthine solution (500 µM) were then added, and the reaction was started by adding 50 µL of XO suspension (23.32 mU/mL), while PBS (50 µL) was added in blank samples. The final concentrations were: xanthine, 50 µM; EDTA, 10 µM and XO, 4.7 mU/mL. The wells were incubated for 30 min at 37 °C, and uric acid production was determined by subtracting the absorbance at 290–295 nm of the sample against that of the blank. Each measurement was performed in triplicate.

#### 2.2.3. Superoxide Radical Quenching

Superoxide (O_2_^•−^) inhibition was evaluated in glycine buffer (6.25 mM, pH 10.1)/acetone medium using an allopurinol-XO generating system coupled with lucigenin assay. All reactants were dissolved in glycine buffer, and the reaction was carried out at 25 °C [30]. Briefly, to glycine buffer (1.275 mL) were sequentially added 0.425 mL of acetone, 300 µL of allopurinol solution (100 µM; final concentration 17.6 µM), 3–25 µL of test compound solution (20 mg/mL in DMSO) and 300 µL of lucigenin solution (100 µM; final concentration 17.6 µM). The test tube was inserted in the luminometer, and reaction was initiated by adding a XO suspension (2 µL, 11 mU/mL). Luminescence was monitored for a total period of 7 min and O_2_^•−^ quenching was calculated by subtracting areas under the curve against blank. Each measurement was performed in triplicate.

#### 2.2.4. Metal Chelating Capacity (MCC)

The MCC of each extract was measured according to a protocol adapted for microplates [23]. Samples (200 µL/well) dissolved in methanol at 0.3–2.4 mg/mL were mixed with 10 µL of aqueous ferrous chloride (2 mM). The mixture was then reacted with 40 µL/well of 5 mM aqueous 3-(2-pyridyl)-5,6-bis(4-phenylsulfonic acid)-1,2,4-triazine (ferrozine; Sigma–Aldrich, Saint-Quentin Fallavier, France) for 10 min at room temperature under vigorous stirring. The absorbance was read at 562 nm, and a calibration curve was constructed using EDTA as a standard. Percent inhibition of [Fe^2+^–ferrozine] complex was calculated against control well absorbance (water instead of extract). The results are expressed as micromoles of EDTA equivalents (EDTAE) per gram of extract weight.

#### 2.2.5. Assay of Proteinase and Lipoxygenase Inhibitory Activities

Anti-inflammatory properties of the extracts were evaluated by their proteinase and lipoxygenase inhibitory activities as described [31]. Briefly, PTA (0.06 mg) was dissolved in 2 mL of Tris HCl buffer (20 mM, pH 7.4) containing a suspension of each extract (100–600 µg/mL). The mixture was incubated at 37 °C for 5 min, and 1 mL of 4% BSA was added. Incubation was prolonged for 20 min, and afterwards 2 mL of 5% trichloroacetic acid was added to the mixture. After centrifugation (3000 rpm, 10 min, 4 °C) the absorbance was read at 210 nm against buffer as blank, and the percentage of proteinase inhibition activity was calculated. Experiments were performed in triplicate and averaged. The anti-inflammatory drug diclofenac (100–300 µg/mL) was used as a standard for comparison.

Lipoxygenase (15-LOX, soybean) inhibitory activity was evaluated using a commercial assay kit (Cayman Chemicals Co; Item No. 760700) according to the manufacturer’s instructions. Solutions of test samples (10 μL) in methanol were added into 96-wells plates together with assay buffer, and the enzyme was added. The reaction was initiated by adding 10 μL of arachidonic acid to the wells and the plate was placed on a shaker for 15 min. Chromogen solution (100 μL) was added to the wells, the plate was then placed on a shaker for 10 min and absorbance was read at 495–500 nm. The flavonoid quercetin (50–100 µM) was used as a standard. Results are expressed as a percentage relative to the activity without the test compound.

#### 2.2.6. DPPH Assay

Experiments were performed according to previous procedures [22,23]. Aliquots of dichloromethane solutions of oil or fractions (50–500 mg/mL) or test compounds (final concentration 0.01–2 mM) were added to 200 µL of a freshly prepared DPPH solution (75 mg/L), and the final volume was adjusted to 300 µL with dichloromethane to obtain a final DPPH concentration of 0.13 mM. The absorbance was read at 517 nm on a microplate reader 3 min after sample addition. Experiments were performed in triplicate and averaged. The DPPH inhibition was calculated as follows:

Inhibition (%) = [100 × (A_0_ − A)/A_0_] (1)

For each compound, concentration resulting in 50% DPPH scavenging (IC_50_ value) was calculated using Prism 6 software (GraphPad, San Diego, CA, USA).

#### 2.2.7. Total Peroxyl Radical-Trapping Potential (TRAP) Assay

The experimental protocol of the TRAP assay was previously reported [30]. Briefly, AAPH (150 mM) was dissolved in 10 mL of Tris HCl buffer solution (100 mM, pH 7.4), and the solution was kept at 37 °C for 30 min to initiate thermal radical formation. A luminol stock solution (1 mM) was prepared in 50 mL Tris HCl buffer and kept in darkness at 4 °C. A 78 µM, working luminol solution was prepared before each assay by dilution in Tris HCl buffer. Samples for assays were prepared as follows: 2 mL of AAPH (final concentration, 100 mM) and 500 μL of luminol solution (final concentration, 13 µM) were introduced in a 3 mL tube; then, 500 μL of pure acetone (blank) or solution of the tested oil or extract in acetone (0.005–0.2 mg/mL) or DMSO (test compounds: 5–300 µM) were added and the mixture vortexed for 5 s before luminescence analysis. A calibration curve was obtained in the same conditions using the antioxidant Trolox (1–40 μM) as a standard.

Quantification of radical scavenging capacity was performed by integrating the area under the curve (AUC) for the sample and blank solutions, and the results are expressed as Trolox Equivalents (TE) using Equation (2):

TE = ([Trolox] × ΔAUC _Sample_)/([Sample] × ΔAUC _Trolox_) (2)

where ΔAUC _Sample (Trolox)_ = AUC _Blank_ − AUC _Sample (Trolox)_.

### 2.3. Biological Experiments

#### 2.3.1. Cell Culture and Reagents

Doubly distilled deionized water was used throughout, and solutions were passed through a 0.2 µm Millipore filter before use. Culture media Dulbecco’s modified Eagle medium (DMEM), Roswell Park Memorial Institute medium (*RPMI*), Eagle’s minimal essential medium (MEM), GlutaMax^®^ and fetal calf serum (FCS) were from Life Technologies. Other reactants such as 1,1,3,3-tetramethoxypropane (TMP), 3-(4,5-dimethylthiazol-2-yl)-2,5-diphenyltetrazolium bromide (tetrazolium dye, MTT) and Triton X-100 were from Acros Organics or Sigma-Aldrich.

The microorganisms *M. tuberculosis* H37 RV (ATCC 27294, France) and Staphylococcus aureus (ATCC 6538, France) strains were a gift from IRD-Aix Marseille University and were cultured according to [12] and [32], respectively. Murine 3T3 fibroblasts (ATCC-LGC Promochem, Molsheim, France) were maintained in the culture at 37 °C in a 5% CO_2_-humidified atmosphere in DMEM containing 1% glucose and supplemented with 10% FCS, 100 U/mL penicillin and 100 mg/mL streptomycin essentially as described earlier [33]. Cells were plated in 24-well dishes, and the medium was replenished every 2 days until confluency, which was checked by microscopic observation. A549 human lung carcinoma cells were cultured at 37 °C in an atmosphere of 5% CO_2_ in RPMI supplemented with 10% FCS, 100 U/mL penicillin, 100 µg/mL streptomycin and 2 mM GlutaMax^®^ [23]. Flasks containing confluent cells were trypsinized, and cells were seeded in 96-well plates at a density of 2 × 10^4^ cells per well in complete RPMI and incubated for 48–72 h with test compounds.

#### 2.3.2. Antimicrobial and Anti-Mycobacterial Activity

The antimicrobial activity was evaluated against the microorganism *S. aureus* (ATCC 6538, France) according to the disk-diffusion agar method [34]. Briefly, 20 µL per aliquots of each compound (stock solutions of 1 mg/mL in DMSO) were impregnated on a paper disc. After 24 h incubation at 37 °C, the diameter of the zone of inhibition was determined. The antibiotic oxacillin (20 µL of a 1 mg/mL solution in DMSO) was used as the positive control.

The anti-mycobacterial activity was assessed against *M. tuberculosis* H37 RV using the colorimetric Microplate-based Alamar Blue assay (MABA) [12]. For each test compound, the minimum inhibitory concentration (MIC), which is the lowest concentration inhibiting the visible growth of the microorganism after 24 h incubation at 37 °C, was expressed in µg/mL from at least 3 independent experiments. The antibiotic streptomycin was studied for comparison.

#### 2.3.3. Cytotoxicity Assays

The cytotoxicity of TO, resin fractions and test compounds were evaluated on 3T3 fibroblasts and A549 cells using the MTT assay performed in microplates [30,33]. Briefly, following 48 h incubation at 37 °C with TO or fractions (0.001–1000 µg/mL), MTT in PBS 1X (+/+) (5 mg/mL) was added to the medium (final concentration, 0.5 mg/mL) and incubation was extended for 4 h at 37 °C. Then, medium was removed and DMSO (100 µL) was added to each well and incubated for 15 min with agitation in a shaking incubator. The conversion of MTT to a purple formazan precipitate was monitored at 570 nm. The inhibition of cell viability (IC_50_) was calculated using linear regression calculations from at least 5 different concentrations for each sample.

#### 2.3.4. Experiments on 3T3 Cells Exposed to tBuOOH

Aliquots of test compounds (10–20 µL) diluted in phenol-red-free DMEM medium containing 1% glucose were transferred into the culture wells pre-filled with the appropriate volume of medium to reach a final volume of 0.5 mL/well. After 2 h incubation, oxidative stress was induced by incubating the cells with *t*BuOOH (0.40 mM) for 4 h [35]. Then, the supernatants were sampled for cytoplasmic LDH release, and the adherent cells were preincubated for 2 h in MEM containing neutral red (50 µg/mL) and 2% FCS. Cell viability was determined in a microplate reader by monitoring the absorption difference between 560 and 620 nm. In the second set of wells, both LDH release and cellular MDA-TBA contents were assayed in supernatants and scraped cells, respectively. LDH activity was assessed at 340 nm using a commercial detection kit (Biolabo, Maizy, France). Protein measurement was performed in 4 out of 24 randomly selected wells. To determine the total LDH content (i.e., the sum of the enzymatic activity in the lysate and the culture medium), 1% Triton X-100 was added to randomly selected wells of untreated cells. Then, the cells were scraped and homogenized in serine-borate buffer and the MDA content of the suspension was measured by HPLC using UV-visible detection according to a previously reported procedure [22]. Results were compared to control cells treated with DMEM + 0.1% DMSO. Data represent 6 independent experiments made in duplicate for each test compound and resin concentration and are expressed in arbitrary units/mg of protein.

#### 2.3.5. Experiments on UV Irradiated Rat Skin Slices

Skin samples were obtained from the back of six normal Sprague Dawley rats (CERJ, Le Genest St Isle, France) weighing 100–150 g within 2 h after sacrifice by intraperitoneal injection of pentobarbital (120 mg/kg; Ceva Santé Animale, Libourne, France). Before sacrifice, animals were used for other experiments than the present study (agreement for animal experimentation APAFiS 2015102022108840). Animal care was performed according to the rules of the European Union Council (Revised Directives 2010/63/EU). Aix Marseille University and the CNRS have a license for animal housing and experimentation (agreement C13-055-06), and the study was under the supervision of a vet at the CNRS.

Skin samples were shaved, rinsed first in water and then three times in 125 mM aqueous KCl, dried, cut into slices of about 15 × 10 × 0.5 mm size and kept for 1 h on agar plates at 4 °C [21]. Afterwards, skin samples were placed at the surface of a filter paper soaked with aqueous KCl to avoid any dehydration and maintained for 10 min at 37 °C. Each test extract (50 µg) was emulsified with 100 µL of olive oil and spread on the surface of the skin slice [16]. They were allowed to stabilize for 1 h at 37 °C before exposure for 1 h to a UVA/UVB solar simulator (150 W, 280–400 nm; Lot-Oriel, Palaiseau, France) in the conditions of a phototoxic dose [22]. Following irradiation, the slices were separated into two groups.

In the first group, the slices were first homogenized in 5 volumes of aqueous KCl (125 mM). Afterwards, homogenates (0.25 mL) were added 0.7 mL of 5% trichloroacetic acid and 50 μL of 0.2% butylated hydroxytoluene, the mixture was incubated for 60 min at 95 °C, quickly cooled to room temperature and centrifugated (1000× *g* at 4 °C), and the supernatant was mixed with an equal volume of saturated aqueous TBA solution and re-incubated for 60 min at 95 °C. After cooling, each sample was extracted with 2 mL of butanol/pyridine, 15/1, (*v*/*v*) and the absorbance of the MDA-TBA complex was read at 532 nm. A calibration curve was obtained from standard MDA-TBA samples prepared by using 0.25 mL of 0.1–15 mmol/L of TMP solutions instead of skin homogenate. Data are representative of 6 independent experiments for each sample and are expressed as µmol/mg protein.

In the second group, each skin slice was covered with 0.5 mL of PBS before receiving one 20-µL aliquot of the nitrone 5-(diethoxyphosphoryl)-5-methyl-1-pyrroline *N*-oxide (DEPMPO) [36] (final concentration, 15 mM) in PBS immediately after irradiation was switched off. Slices were incubated in the dark for 15 min at room temperature to allow for spin adduct build-up. Afterwards, the supernatants were transferred into cryotubes and immediately stored in liquid N_2_ for delayed electron paramagnetic resonance (EPR) analysis.

#### 2.3.6. EPR Experiments

Samples for EPR were sequentially thawed, placed into calibrated 50 µL glass capillaries and single scanned for their spin adduct content using a Bruker ESP 300 spectrometer (Karlsruhe, Germany) operating at X-band (9.81 GHz) with a 100-KHz modulation frequency. Spectra (magnetic field resolution, 2048 points) were recorded at room temperature 1 min after thawing of the sample using the following parameters: microwave power, 10 mW; modulation amplitude, 0.63 G; receiver gain, 5 × 10^5^; time constant, 20.48 ms; and scan rate, 0.7 G/s for a sweep width of 120 G. Computer simulations of the EPR signals were carried out assuming they consisted of varying mixtures of DEPMPO-OH, the DEPMPO/hydroxyl radical (HO•) spin adduct (giving an 8-line spectrum with coupling constants a_N_ = 14.0 G; a_H_ = 13.3 G and a_P_ = 47.2 G) and DEPMPO-OOH (the DEPMPO/O_2_^•−^ spin adduct), the main *trans*-DEPMPO-OOH diastereoisomer of which existing as two fast-exchanging rotamers (65:35%), both giving 12-lines spectra with a_N_ = 13.3(12.9) G; a_H_ = 12.2(8.6) G and a_P_ = 53.9(43.0) G [22,36] (see structures in Scheme S1). Relative spin adduct concentrations were obtained by double integration of the simulated signals using WinSim software [37].

#### 2.3.7. Statistical Analysis

All values are expressed as mean ± SD or SEM for the indicated number of independent experiments. Differences were analyzed by one- or two-way analysis of variance (ANOVA) followed by appropriate a posteriori tests. Intergroup differences were considered significant at *p* < 0.05.

## 3. Results and Discussion

### 3.1. Chemistry, HPLC, TPC and TFC Analyses

Tamanu oil was obtained from crushed air-dried mature seeds of *C. inophyllum* as a green oil with a heavy texture. As a rather high acid index value (14–40) was found for the oil (data not shown), the presence of amounts of organic acids was expected. After filtration, fractionations were performed to give the methanol (MeTO) and ethanol (EtTO) soluble extracts (Scheme 1). Ethanol was preferred to other solvents (e.g., *n*-hexane or ethyl acetate) for more efficiently removing fatty acids, triglycerides and tannins [4], whereas methanol is highly efficient for recovering amounts of polar phenolics and flavonoids [20,23]. Then, EtTO was further separated according to a multistep procedure into neutral (NTR) and acidic (ATR) fractions, while MeTO resin was kept unchanged for further analysis. A methanolic extract of EtTO was prepared to yield the MeETR fraction.

**Figure 1:**
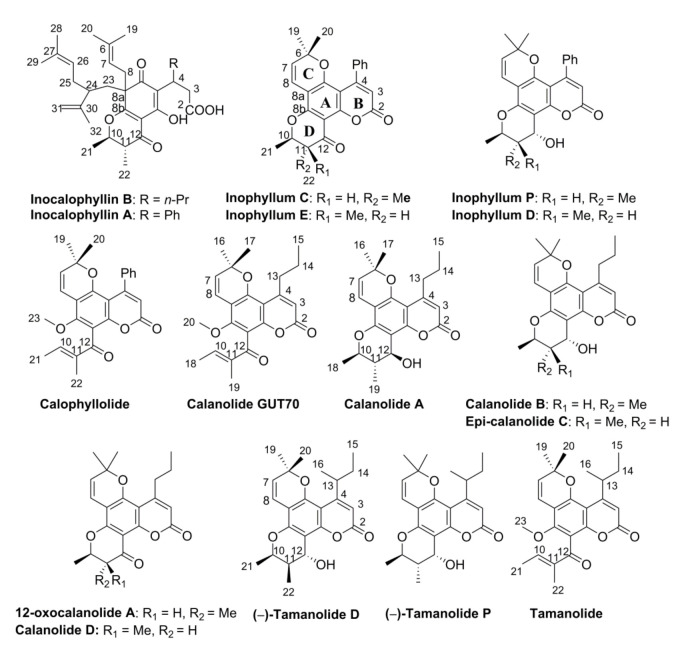
Structure of the secondary metabolites belonging to the pyranocoumarin family isolated from NTR and ATR fractions by low-pressure column chromatography and HPLC separation. ^1^H-NMR, ^13^C-NMR and MS spectra are reported in Supplementary Information.

LPLC on NTR and ATR resins led to ten and four subfractions, respectively. Fractions of middle polarity containing substances with keto or hydroxyl groups, as indicated by TLC analysis (see Methods), were then submitted to further HPLC separation. In our hands, fifteen pyranocoumarin derivatives were identified, fourteen from the NTR fraction: inophyllums (C, D, E, P), calophyllolide, calanolides (A, B, D, GUT 70, epi-calanolide C, 12-oxacalanolide A) and three tamanolides (tamanolide, tamanolides D and P), and one from the ATR fraction, i.e., inocalophyllin B (Figure 1). The structure of each phytoconstituent was assigned according to its ^1^H-NMR spectrum, MS analysis and ^13^C-NMR for some products (see Supplementary Information) and compared to literature data if the same metabolites were obtained previously from other natural sources or by chemical synthesis. For derivatives obtained with at least 97% purity (by HPLC), optical activities were measured (see Materials and Methods).

Inophyllums C, D, E and P have been previously reported in *C. inophyllum* leaves or limbs from Malaysia [10,38]. Inophyllum C was also found in *C. inophyllum* nuts from Madagascar and leaves of *C. inophyllum* from French Polynesia [39]. Inophyllum E was isolated in the *C. inophyllum* oil from Fiji Islands [7]. However, the present work is the first detailed report of inophyllums D and P isolated from *C. inophyllum* oil and the first report of inophyllums C and E in the oil from French Polynesian species.

Moreover, three other calanolides were isolated as pure compounds from EtTO extract, i.e., calanolides A and D, and 12-oxocalanolide. Of these three compounds, only calanolide A has been already isolated in Calophyllum lanigerum [6] and Calophyllum brasiliense [40]. In *C. inophyllum*, only calanolide GUT 70 has been previously reported from Madagascar species [41], being later isolated from the stem bark of *C. brasiliense* [42]. However, to the best of the authors’ knowledge, this is the first time that calanolide GUT 70 has been ever identified in *C. inophyllum* from French Polynesia, based on NMR (^1^H and ^13^C) and UV data (see Section 2.1.6 and Supplementary Information), in good agreement with those reported from other natural species ([6,40] or from chemical synthesis [43]. Furthermore, this study also reports on the first identification of calanolide D and 12-oxocalanolide A from a natural source, both compounds being previously obtained by chemical synthesis [26,29]. Calanolide D was claimed to have been isolated from *C. lanigerum* [6], but the structure of the molecule was reassigned later as being a regioisomer, pseudo-calanolide D, in which the pyran ring positions are inverse with respect to the coumarin moiety [9,26,29]. Moreover, the synthesis of both pseudo-calanolides D and calanolide D in their racemic forms was reported, and the authors highlighted small differences in their ^1^H-NMR spectra, mainly affecting the C-8 pyran ring protons [26]. In the present work, according to ^1^H-NMR spectra (see Supplementary Information), the molecule isolated from *C. inophyllum* corresponds to the calanolide D structure as shown in Figure 1. Indeed, the ^1^H-NMR doublet at 6.65 ppm was unambiguously attributed to the *H*-C-8 belonging to the pyran ring. Unfortunately, the relatively low purity of the sample (96% by HPLC) could not allow the determination of the dextro- or levogyre nature of the enantiomer.

The spectroscopic parameters of the compound assigned to 12-oxocalanolide A were in good agreement with those obtained by organic synthesis [26,44]. Of interest, two other calanolides were observed as minor constituents, i.e., calanolide B in Fraction P (which contained also inophyllum P and tamanolide P) and the epi-calanolide C in mixture with tamanolide D and inophyllum D in Fraction D. Again, the ^1^H-NMR data were in good agreement with the literature [6,26,45].

According to their HPLC purity and availability requirements, ten isolated C. *inophyllum* oil metabolites and the fully characterized multi-components Fraction P were selected for further antioxidant and biological evaluations, together with the DTO, MeTO, MeETR, EtTO, NTR and ATR extracts. First, it was considered interesting to quantitate the TPC and TFC values in crude oil and extracts. The data reported in Table 1 show that using ethanol as starting material for fractionation significantly favored the recovery of phenolic compounds, i.e., a 3–6 fold increase in TPC values was found in EtTO, ATR and especially NTR extracts, and a 2–7 fold increase in TFC values was found in EtTO, NTR and especially ATR extracts, as compared to the crude oil. Regarding TPC and TFC values methanol was globally a less efficient extraction solvent in both MeOH and MeETR extracts (Table 1). No measurable amounts of phenolic and flavonoid derivatives were found in the de-resinated DTO fraction.

In Calophyllum oil, TPC was considered to reflect the content in reducing compounds, including benzoic acid derivatives [46], while its resinous fraction can contain up to 5% of polyphenols [47]. Analysis of the flavonoid content using AlCl_3_ complexation was reported to be mainly selective for flavonoids bearing C-3-OH (flavonols) and C-5-OH substituents, and for compounds bearing vicinal hydroxyl groups on the B ring, such as luteolin [48]. Biflavonoids have been also mentioned in TO [3]. Aluminium chloride complexation may also occur at other sites on flavonoids making more complex the interpretation of the TFC assay owing to the existence of several absorption wavelengths.

Altogether, the results here highlight that the resin part of TO is characterized by high levels of phenolic and flavonoid derivatives and that the ethanol-soluble extracts NTR and ATR would likely contain most of the functionalized secondary metabolites.

### 3.2. In Vitro Antioxidant and Anti-Inflammatory Activities

Antioxidant and anti-inflammatory activities of resin extracts and selected isolated compounds were evaluated by a series of assays relevant to ROS and ROS precursors that differ in their endpoints and mechanisms or implicated biological targets.

#### 3.2.1. Xanthine Oxidase Inhibition, Superoxide Quenching and MCC

To limit oxidative stress, quenching and/or inhibiting O_2_^•−^ formation could be of relevance for the medicinal use of TO and resins. XO inhibition by natural polyphenols, phenolic acids and flavonoids [30,49] or coumarins derivatives [8] has been reported. In the present work, ATR and NTR exhibited higher activities than EtTO (IC_50_~100 µg/mL), with IC_50_ values ranging from 71 to 93 µg/mL (Table 1). Fraction P (IC_50_~120 µg/mL) and the methanolic extracts MeTO and MeETR (IC_50_ > 150 µg/mL) were significantly less potent, whereas the crude oil was poorly active (IC_50_~950 µg/mL) and the de-resinated DTO was inactive. It has been reported that XO inhibition is strongly enhanced by the presence of hydroxyl substituents at positions 6 and 7 of the coumarin benzo moiety, and possible interaction of coumarins with the molybdenum domain of the enzyme was investigated [8]. Among the coumarins identified in the TO extracts, some of them bear hydroxyl groups, but not in the optimal position to bind the XO active site. Hence, XO inhibition efficacy found here, i.e., ATR > NTR > EtTO > Fraction P > MeTO ≈ MeETR, could be directly correlated to TFC. However, the possible effects of other metabolites not yet identified in the resins remain to be investigated.

Superoxide quenching capacity of TO and resins was evaluated in acetone by monitoring the inhibition of lucigenin luminescence from an allopurinol-XO O_2_^•−^ generator. Table 1 shows that extracts and fraction P that demonstrated the best XO inhibition property were also much stronger O_2_^•−^ scavengers (i.e., 5–9–fold) as indicated by the lower IC_50_ values ranging from 14 to 25 µg/mL.

The MCC value has been reported as an index of the ability to form complexes with Fe^2+^ to inhibit iron-induced damage [50]. Expectedly, the best activity in this assay was exhibited by phenolics- and flavonoids-enriched NTR and ATR at a level comparable to that of, e.g., quercetin.

#### 3.2.2. Anti-Inflammatory Properties of TO Extracts and Subfractions

Inflammation plays a crucial role in the pathogenesis of several diseases and is characterized by the production of pro-inflammatory mediators and infiltration of various types of cells. Due to their antioxidant properties found above, it was interesting to investigate the potential of TO resins as in vitro anti-inflammatory agents on the two key enzymes, PTA and 15-LOX.

The results obtained in vitro in presence of serum albumin showed that the percentage of PTA inhibition by ATR, NTR and EtTO extracts ranged 62–72% and were nearly equal to the standard drug diclofenac (64%) at the same concentration of 150 µg/mL (Table 1). Methanolic extracts were less potent (29–31% inhibition), and the crude oil was found poorly active. Again, DTO was found to be inactive, showing that the resin is an essential component of TO biological activities.

In cells, 15-LOX catalyzes the oxidation of unsaturated fatty acids such as arachidonic acids to yield hydroperoxy fatty acids which could, in turn, mediate inflammation processes and trigger oxidative stress. It has been reported that flavonoids act efficiently as lipoxygenase inhibitors, both by scavenging peroxyl radicals and inhibiting lipid peroxidation [51].

Here, the standard quercetin induced a strong inhibition of 15-LOX (83% at 100 µg/mL), an effect potentially linked to the presence of hydroxyl groups in its structure, which could both interfere with the enzyme and scavenge free radicals [51]. Again, the two fractions NTR and ATR, rich in phenolic compounds and flavonoids, were found to be highly effective regarding enzyme inhibition (45–60%). Interestingly, the fraction P containing 70% of inophyllum P was also found to be highly effective against 15-LOX (56% inhibition), a result in line with previous findings on inophyllums D and H isolated from Calophyllum symingtonianum [52]. Inophyllum P (see Figure 1), as well as inophyllums D and H, contain an OH group in its structure.

#### 3.2.3. DPPH and TRAP Assays

The DPPH assay, an index of global free radical reducing activity, was performed in dichloromethane. The TRAP assay, which evaluates the capacity to scavenge peroxyl radicals, was performed in aqueous/acetone medium. The results are reported in Table 2.

Despite sesame oil being relatively rich in antioxidants, including sesamol [21,22], TO was found to be more active in both DPPH and TRAP assays, suggesting that the oil contains more lipophilic antioxidants than sesame oil. Moreover, the antioxidant properties of TO may rely mainly on the resin part since the de-resinated DTO was found to be inactive. Remarkably, the crude oil was found ~30-fold less active than resin extracts and subfractions, with ATR being the most efficient. The DPPH scavenging capacity of the secondary metabolites isolated from NTR and ATR was also evaluated. In this test, the compounds were as follows: strongly active (inophyllum P > fraction P), moderately active (inophyllum E > inocalophyllin B > inophyllum C > tamanolide D), slightly active (calophyllolide ≈ calanolide GUT 70 > calanolide A) and inactive (12-oxocalanolide A, calanolide D). Inophyllum P, bearing a chromanol hydroxyl group on the C-12 of the D-ring, is 20–30 times more active than inophyllums E and C, which lack this function. However, these latter exhibited better activity than calanolide D and 12-oxocalanolide A.

**Figure 2:**
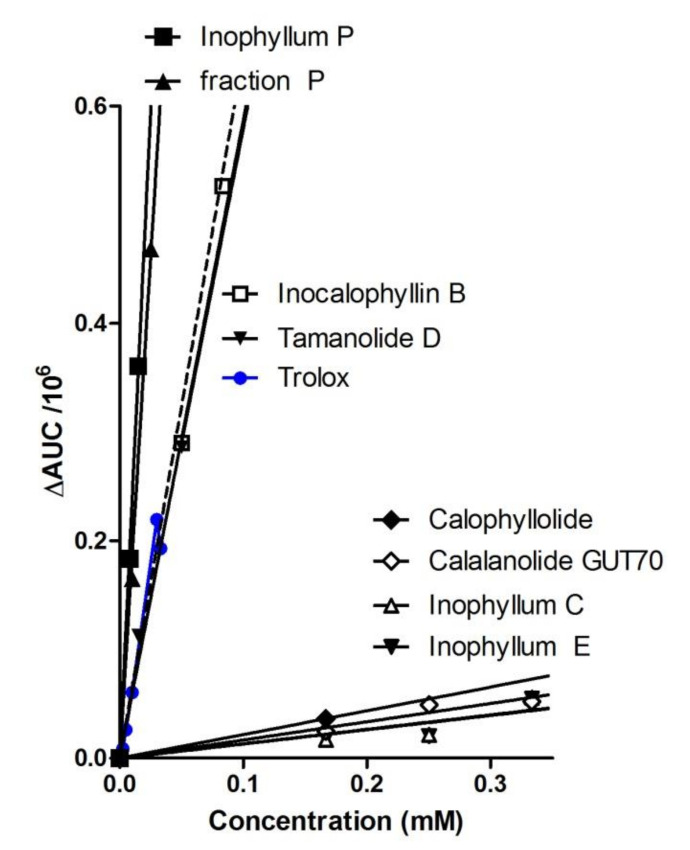
Superoxide scavenging capacity of TO extracts metabolites by TRAP luminescence assay. Linear correlations plots are expressed as area under the curve (ΔAUC) versus compounds concentrations. In fraction P, inophyllum P accounts for ~70% in weight.

In the TRAP assay, all extracts, especially ATR, were found to be much more efficient than crude TO, which yielded at least 4-fold lower TE values. Among the isolated compounds a strong antioxidant activity was found for inophyllum P and Fraction P, followed by tamanolide D and inocalophyllin B. The other metabolites were found to be poorly active in the assay, unrelated to the calculated lipophilicities (as AlogP values). Figure 2 shows the correlation of the measured ΔAUC with the sample concentration in the TRAP assay for seven selected compounds and fraction P, evidencing the strong capacities of inophyllum P, fraction P and inocalophyllin B in peroxyl radicals scavenging. This has been proposed to be a possible antioxidant mechanism of coumarins besides enzyme inhibition and chelation of pro-oxidant catalytic metals [53].

Several coumarins extracted from *C. inophyllum* exhibited rather strong antioxidant properties in the TRAP and DPPH assays despite they do not bear a catechol structure nor phenol groups, both structural patterns known to confer free radical scavenging power [53,54]. Interestingly, two structural patterns occur in these active compounds: (i) a 4-chromanol moiety, such as in inophyllum P, tamanolide D, calanolide A and tamanolide D contained in fraction P, and (ii) other potential radical scavenging groups such as non-phenolic conjugated OH groups (in inocalophyllin B) or α,β-unsaturated ketones (present in each coumarin scaffold). The high activity observed for inophyllum P, close to that of the phenolic standards (Table 2) and of fraction P, containing tamanolide P, calanolide B and inophyllum P (all three compounds bearing the chromanol part) could rely on two frameworks, the chromanol and the coumarin patterns in the structure. Therefore, inophyllum P is about four times more efficient than Trolox in the TRAP assay. Inocalophyllin B also exerted a significant TRAP activity, which could be related to the presence of (i) the ketone conjugated OH group due to the saponification of the lactone B-ring, and (ii) two prenylated side chains [54].

Inophyllums C and E, calophyllolide and calanolide GUT70, bearing a chromanone moiety instead of a chromanol, are weakly active. The DPPH inactive calanolide D and 12-oxocalanolide A, not featuring those potential antioxidant patterns and bearing a chromanone moiety, have not been tested in the TRAP assay.

Given all the above aspects dealing with ROS inhibition, it has been demonstrated here that extracting crude TO with an appropriate solvent such as ethanol is a very powerful route toward a series of EtTO-derived fractions enriched in compounds conferring substantially increased antioxidant/free radical scavenging and anti-inflammatory capacities. Among these, neutral resin NTR contains the major part of the identified molecules, inophyllum P being one of the most abundant (about 16% of EtTO isolated products) and the most efficient in DPPH and TRAP assays. In this latter regard, the acidic resin ATR was even more interesting because of the presence of the strong antioxidant inocalophyllin B in a relatively high yield (about 21% of EtTO-isolated products).

### 3.3. Antimicrobial and Anti-Mycobacterial Activity

The antimicrobial activity of TO fractions was first screened against *S. aureus* using the disk diffusion assay, with oxacillin used as a positive control. The results are reported in Table 3.

At 20 µg per disc, all ethanol-derived extracts and isolated fractions exhibited significantly better antimicrobial activities than TO and MeTO, which were found inactive. The most active fractions were NTR and ATR and, to a lesser extent, EtTO, however, still to a notably less efficient than oxacillin. From the NTR fraction, inophyllum P and fraction P exhibit significant activity as well as calophyllolide and 12-oxocalanolide A. Inocalophyllin B from ATR fraction was also found active against *S. aureus*, and its resulting antimicrobial was roughly similar to that of ATR. The antimicrobial efficiency of several other constituents from the genus *Calophyllum* has been reported against *S. aureus* but not against other microorganisms [11]. This point remains to be further investigated using the present TO resins and fractions on several other strains.

The activity of compounds against strain H37Rv of *M. tuberculosis* was evaluated by determining the MIC defined as the lowest concentration inhibiting at least 99% of the bacterial population present in the medium at the beginning of the assay. The results indicated that EtTO, ATR, and especially NTR and pyranocoumarins exhibited significant antimicrobial activity. Interestingly, ATR was found less cytotoxic than EtTO, NTR and all other compounds, including inocalophyllin B, both on cancer A549 cells than normal 3T3 fibroblasts. Likely because of its high lipophilicity and the weak proportion of bioactive constituents in its lipid phase, TO was found both cytotoxic at low concentrations and poorly efficient as antimicrobial agent.

Coumarin has been shown to exhibit antimicrobial activity against Gram-positive and Gram-negative bacterial strains, a property attributed to its aromaticity and lipophilicity, which can allow it to cross the bacterial wall and interact with the cell membrane [55,56,57]. The presence of a pyran ring in the coumarin structure does not necessarily enhance the antibacterial activity, but the presence of OH groups might be favorable. On the other hand, phenolic compounds often exhibit antimicrobial properties, and among their possible mechanisms of action, it has been suggested that the bacterial membrane potential can be destabilized by OH groups [58]. Metal deprivation or enzyme inactivation by phenolics have also been evoked [59]. Here, NTR, which is rich in phenolic derivatives as indicated by its TPC value (Table 1) and gathers amounts of OH-bearing pyranocoumarins (i.e., inophyllum P, tamanolides D and P, and calanolide A) exhibited a higher antibacterial efficiency against both *S. aureus* and *M. tuberculosis* than ATR. This latter fraction contains, however, the prenylated inocalophyllin B, which appears to be mainly responsible for ATR activity against *S. aureus*. Then, even if bactericidal activity can also involve ROS production [55], bioactive molecules can share some structural features supporting both antioxidant and antibacterial activities through different mechanisms [60].

Owing to their biological properties, TO resin extracts could lead to further development of new sources of naturally occurring anti-infectious agents.

### 3.4. Biological and EPR Evidence of the Protective Effects of Tamanu Oil Derivatives against Oxidative Stress

**Figure 3:**
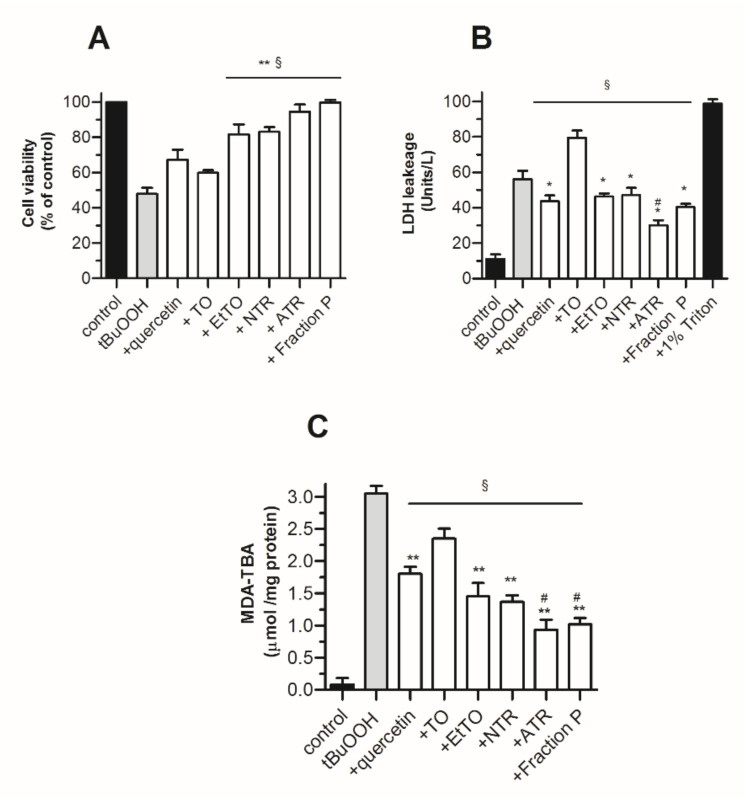
Protective effect of TO extracts and metabolites against *t*BuOOH-induced cytotoxicity in 3T3 fibroblasts (1 × 10^6^ cells/mL). Cell viability and oxidative stress were assessed after 4 h of incubation in the absence or presence of test compounds at 15 µg/mL, by (**A**) LDH leakage in culture supernatant, (**B**) neutral red-based viability assay, (**C**) MDA-TBA levels. Quercetin was used at 3 µg/mL. Values represent means ± SEM (*n* = 8). Statistics: * *p* < 0.05 and ** *p* < 0.01 vs. TO, ^#^ *p* < 0.05 vs. EtTO and ^§^ *p* < 0.01 vs. *t*BuOOH, by one-way ANOVA (*p* < 0.01) followed by Newman–Keuls test.

The protective effect of resins and metabolites on 3T3 fibroblasts undergoing oxidative stress was evaluated using three indexes: (i) the amount of cytosolic LDH released from damaged cells, (ii) cell viability using the neutral red assay and (iii) oxidative stress status through cellular MDA-TBA formation. Oxidative stress was induced by exposing the cells for 4 h to 0.4 mM *t*BuOOH, a concentration close to its reported IC_50_ value in fibroblasts [35]. At the non-cytotoxic dose of 15 µg/mL (Table 3), the resins EtTO, NTR, ATR and Fraction P significantly prevented LDH leakage in culture supernatants, improved cell viability and protected cells against MDA formation (Figure 3). Despite its low solubility in aqueous medium, the flavonoid antioxidant quercetin at 3 µg/mL afforded similar protection against cell damage and oxidative stress but only poorly prevented the known *t*BuOOH induced ROS dependent apoptotic effect [35]. The finding that treatment with the crude oil induced a slight LDL leakage illustrates the benefit of using TO derived resins rather than TO itself for best protecting the cells.

Finally, the photo-protective efficacy of resins in simple ointment-based applications was investigated on rat skin slices using a model widely used in pharmacological and dermatology research. Crude TO, EtTO and derived resins were applied topically onto the skin slices as olive oil emulsions [16] before a phototoxic dose of UV light was applied for 1 h.

**Figure 4:**
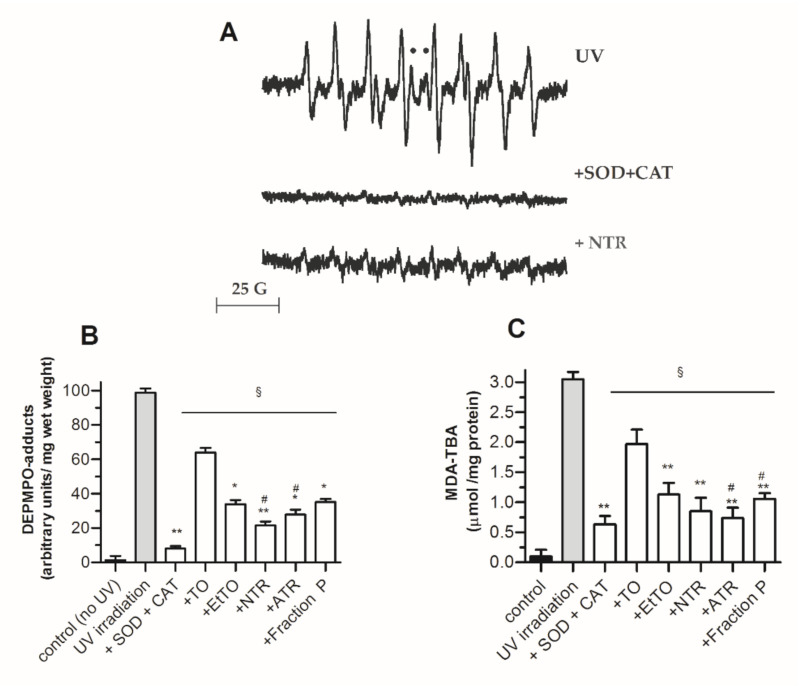
Protective effect of TO extracts and metabolites against UV irradiation (1 h)-induced damage in rat skin slices. (**A**) Typical electron paramagnetic resonance (EPR) signals of mixtures of DEPMPO-OOH + DEPMPO-OH in the supernatants of post-irradiated skin slices incubated with aqueous DEPMPO (15 mM). SOD, superoxide dismutase (10 U/mL); CAT, catalase (5000 U/mL); NTR, neutral resin subfraction; ●, DEPMPO-OH central lines. (**B**) Total DEPMPO adducts levels. (**C**) MDA-TBA levels. Values represent means ± SEM (*n* = 8). Statistics: * *p* < 0.05 and ** *p* < 0.01 vs. TO, ^#^ *p* < 0.05 vs. EtTO and ^§^ *p* < 0.01 vs. UV-irradiated untreated slices, by one-way ANOVA (*p* < 0.01) followed by Newman–Keuls test.

First, spin trapping was applied, an indirect detection technique in which transient free radicals are allowed to form EPR-detectable adducts with nitrones for the purposes of identification and quantification [36]. When samples from untreated post-irradiated skin slices were dark-incubated for 15 min in a milieu supplemented with the nitrone DEPMPO (15 mM), strong EPR signals were detected in the supernatant, the simulation of which was consistent with a mixture of DEPMPO-OOH (~80%) and DEPMPO-OH (~20%) (upper trace in Figure 4A). Almost complete inhibition of this signal by a SOD (10 U/mL) + CAT (5000 U/mL) mixture (Figure 4A, middle trace) suggested O_2_^•−^ was the primary trapped radical, although other reactive species such as singlet oxygen (a ROS not yielding DEPMPO adducts) are known to form in UV-irradiated cells [22]. Moreover, although direct scavenging of HO• cannot be excluded, DEPMPO-OH adducts seen in Figure 4A may have originated from other HO•-independent mechanisms, such as metal-ion-catalyzed nucleophilic addition of water to the nitrone or reduction of DEPMPO-OOH by glutathione peroxidase [36]. Consistent with their global antioxidant properties investigated above, TO and all derivatives were found to be significant inhibitors of DEPMPO spin adduct formation in post-irradiated skin (as shown for NTR in Figure 4A, lower trace), with a marked better effect seen for EtTO and resins versus TO (Figure 4B).

Expectedly, a similar inhibition pattern among Tamanu compounds was observed with the lipid peroxidation status of irradiated cells, i.e., MDA-TBA content (Figure 4C).

Indeed, wound treatment, either in acute or chronic cases, is one of the most important applications of TO in dermatology [1,2], and it has been found that *C. inophyllum* oils from French Polynesian are among the most efficient [15]. Wound healing is a complex phenomenon, consisting of several successive steps, including haemostasis, inflammation, cell proliferation and remodeling. Presently, it is admitted that ROS are involved in most of these steps, with a crucial role in signaling and antimicrobial action. However, these beneficial effects must be kept under the control of the cell antioxidant pool. In impaired wound healing, such as ulcers occurring in diabetes, venous insufficiency or radiotherapy burns, the healing process is blocked at the inflammatory stage and does not evolve to final cicatrization. In such cases, oxidative stress has been evidenced together with disturbed endogenous antioxidant profile [61,62]. In topical application uses on the skin or mucous membrane wounds, new TO resins could offer a protective coating with anti-inflammatory (tamanolide P), antioxidant (inocalophyllin B, inophyllums P, C and E, and tamanolide D), anti-mycobacterial and antimicrobial (calophyllolide, calanolide A, inophyllums P and E), and fibroblast protective capacities, which can be attributed to ATR and NTR secondary metabolites.

## 4. Conclusions

Here, the biological properties of ethanol-soluble fractions of TO and their corresponding pyranocoumarin constituents were evidenced in a series of in vitro and ex vivo tests. The results demonstrate that (i) the main biological activity of TO can be attributed to the resin part rich in pyranocoumarins and other phenolic and flavonoid derivatives to be determined further; (ii) new resin fractions prepared here from TO extraction are easy to handle and showed significantly better antioxidant, enzyme inhibition, anti-inflammatory and antimicrobial activities than the starting crude TO material, (iii) the improved solubility of these new fractions could be of interest for further development of new pharmacological applications from natural and renewable sources.

## Figures and Tables

**Table 1:** Total phenolic (TPC) and total flavonoid (TFC) contents, xanthine oxidase (XO) inhibition, superoxide (O_2_^•−^) quenching, metal chelating capacity (MCC), proteinase (PTA) and lipoxygenase (15-LOX) inhibitory activities of Tamanu oil (TO), its de-resinated lipid fraction (DTO), methanolic (MeTO and MeETR) and ethanolic (EtTO) extracts and neutral (NTR) and acidic (ATR) subfractions of EtTO.

Compound	TPC ^a^	TFC ^b^	XO Inhibition	O_2_^•−^ Quenching	MCC	PTA Inhibition ^d^	15-LOX Inhibition ^e^
	(mg GAE/g)	(mg RUE/g)	IC_50_^c^ (µg/mL)	IC_50_ (µg/mL)	(µmol EDTAE/g)	(%)	(%)
	***Oils and fractions***						
**Tamanu oil (TO)**	14 ± 2	3.8 ± 0.5	951 ± 6	565 ± 6	21 ± 1	10 ± 1	12 ± 1
**Sesame oil**	12 ± 1	1.5 ± 0.4	831 ± 4	469 ± 5	20 ± 2	12 ± 1	13 ± 3
**DTO**	~0	~0	>1000	>1000	~0	~0	~0
**MeTO**	44 ± 3 *	15.4 ± 0.7 *^#^	152 ± 2 *^#^	22.1 ± 0.5 *^#^	31 ± 3.1 *^#^	31 ± 2 *^#^	25 ± 2 *^#^
**EtTO**	51 ± 2 *	19.4 ± 0.2 *	102 ± 1 *	18.0 ± 0.1 *	45 ± 2 *	62 ± 4 *	31 ± 2 *
**MeETR**	35 ± 2 *	10.9 ± 0.8 *	152 ± 2 *^#^	25.1 ± 0.5 *^#^	30 ± 3 *^#^	29 ± 4 *^#^	19 ± 1 *^#^
**NTR**	79 ± 2 *^#^	22.3 ± 0.3 *	93 ± 2 *^#^	20.7 ± 1.1 *	95 ± 4 *^#^	72 ± 5 *^#^	45 ± 2 *^#^
**ATR**	57 ± 2 *^#^	34.9 ± 0.4 *^#^	71 ± 6 *^#^	17.7 ± 0.1 *	83 ± 5 *^#^	69 ± 3 *^#^	60 ± 4 *^#^
**Fraction P** ^**f**^	–	–	120 ± 8 *	14.1 ± 0.2 *^#^	34 ± 2 *^#^	65 ± 4 *	56 ± 2 *^#^
	***Standard phenolic and flavonoid compounds***						
**Caffeic acid**	–	–	39.5 ± 2.1	0.013 ± 0.06	125± 5	–	45 ± 1
**Gallic acid**	–	–	41.1 ± 1.2	0.022 ± 0.01	90 ± 3	–	28 ± 2
**Quercetin**	–	–	1.52 ± 0.12	0.083 ± 0.06	105 ± 3	–	83 ± 2
**Allopurinol**	–	–	0.13 ± 0.01	–	–	–	–
**Diclofenac**	–	–	–	–	–	64 ± 2	–

Data represent means ± SEM of 3–6 independent experiments performed in triplicate. ^a^ Values are expressed as mg of gallic acid equivalent (GAE) per gram of extract. ^b^ RUE, rutin equivalent. ^c^ IC_50_ = concentration resulting in a 50% decrease in initial activity or concentration. ^d^ Percentage values are indicated for compounds at 150 µg/mL. ^e^ Percent inhibition values are indicated for compounds at 100 µg/mL. ^f^ Fraction P composition: inophyllum P (70%), tamanolide P (20%) and calanolide B (10%). Statistics: * *p* < 0.05 vs. TO and ^#^ *p* < 0.05 vs. EtTO, by one-way ANOVA followed by Newman-Keuls test.

**Table 2:** Antioxidant capacities of Tamanu oil resin extracts, neutral (NTR) and acidic (ATR) subfractions and selected isolated metabolites by the DPPH and TRAP assays.

Compounds	DPPH	TRAP	% by Weight in TO ^a^ or EtTO ^b^	% by Weight in Subfractions	AlogP ^c^
***Oils and fractions***	IC_50_ (mg/mL) ^d^	TE ^e^			
**TO**	432 ± 1.1	67 ± 11			
**Sesame oil**	668 ± 1.2 *	28 ± 6 *			
**DTO**	>800 *	~0	~60		
**MeTO**	40.2 ± 1.1 *^#^	112 + 14 *^#^	18.2 ^a^		
**EtTO**	26.1 ± 0.1	280 ± 12 *	15.7 ^a^		
**NTR**	10.2 ± 1.1 *^#^	172 + 14 *^#^	50.0 ^b^		
**ATR**	5.1 ± 0.4 *^#^	340 ± 11 *^#^	48.3 ^b^		
***NTR metabolites***	IC_50_ (µM)	TE		% by weight in NTR	AlogP
**Inophyllum P**	26.2 ± 3.1	4.11 ± 0.10	0.05	0.121	4.56
**Fraction P** ^**f**^	40.9 ± 1.1	3.57 ± 0.39	0.04 ^e^	0.102	ND ^g^
**Tamanolide D**	779 ± 7	1.36 ± 0.20	0.02	0.038	4.45
**Inophyllum C**	701 ± 9	0.21 ± 0.04	0.042	0.078	4.55
**Inophyllum E**	481 ± 7	0.91 ± 0.04	0.014	0.029	4.55
**Calophyllolide**	~890	0.48 ± 0.08	0.019	0.038	5.3
**Calanolide GUT 70**	~900	0.30 ± 0.03	0.024	0.048	4.96
**Calanolide A**	~950	0.40 ± 0.02	<0.02	0.021	4.18
**Calanolide D**	>1000	ND	0.007	0.015	4.31
**12-Oxocalanolide A**	>1000	ND	0.005	0.011	4.31
***ATR metabolites***	IC_50_ (µM)	TE		% by weight in ATR	AlogP
**Inocalophyllin B**	583 ± 17	1.07 ± 0.06	0.063	0.2	4.7
***Standards***	IC_50_ (µM)	TE			AlogP
**Trolox**	21.2 ± 0.8	1			2.75
**Sesamol**	66.9 ± 3.1	1.34 ± 0.09			0.89
**BHA**	94.9 ± 6.1	3.46 ± 0.20			4.14
**Caffeic acid**	18.1 ± 0.21	2.98 ± 0.14			2.6
**Quercetin**	14.1 ± 0.21	6.31 ± 0.24			1.81

Data represent the mean ± SEM of 3–6 independent experiments performed in triplicate. ^a,b^ Extracts and metabolites content are expressed as a percentage by weight (in g/100 g) of ^a^ TO or ^b^ EtTO. ^c^ Determined using the Virtuel Computational Chemistry Laboratory software at: ALOGPS 2.1 homepage. Available online: (http://www.vcclab.org/lab/alogps/). (accessed on 3 July 2020) ^d^ IC_50_ defined as the concentration resulting in a 50% decrease in the initial DPPH concentration. ^e^ Data are given in Trolox equivalent (TE). For oils and resin fractions, the reported TE values represent the activity of a solution at 1 g/L relative to 1 µM Trolox. For metabolites and standards compounds, TE values represent the activity of a 1 µM-solution relative to 1 µM Trolox. ^f^ Inophyllum P accounts for ~70% in fraction P. ^g^ ND, not determined. Statistics: * *p* < 0.05 vs. TO and ^#^ *p* < 0.05 vs. EtTO, by one-way ANOVA followed by Newman–Keuls test.

**Table 3:** Antimicrobial (*S. aureus*) and antimycobacterial (*M. tuberculosis* H37 RV) activities and cytotoxicity on murine 3T3 fibroblasts and human lung carcinoma cells A549 of Tamanu oil (TO) extracts, subfractions and selected metabolites.

Compounds	Microorganisms			
	*S. aureus*	*M. tuberculosis*	3T3 Fibroblasts	A549 Cells
	*Diameter* (mm) ^a^	MIC (μg/mL) ^b^	IC_50_ (μg/mL) ^c^	IC_50_ (μg/mL)
**TO**	<5	>150	~20	~10
**MeTO**	<5	>150	120 ± 3	50.1 ± 2.6
**EtTO**	13 ± 1 *	17.3 ± 0.2 *	108 ± 4	25.6 ± 0.9 *
**NTR**	23 ± 3 *^#^	2.8 ± 0.3 *^#^	80 ± 8	17.7 ± 2.1 *^#^
**ATR**	19 ± 2 *^#^	10.2 ± 0.5 *^#^	150 ± 6	94.7 ± 3.3 *^#^
**Fraction P** ^**d**^	19 ± 2 *^#^	7.5 ± 0.2 *^#^	89 ± 8 *^#^	25.5 ±2.1 *^#^
**Inophyllum P**	17 ± 1 *^#^	5.7 ± 0.3 *^#^	92 ± 4 *	11.3 ± 0.6 *
**Inophyllum E**	14 ± 2 *	>15	120 ± 9 *	44.8 ± 2.2 *^#^
**Tamanolide D**	12 ± 2 *	5.1 ± 0.3 *^#^	46 ±5 *^#^	9.3 ± 1.2 *
**Calophyllolide**	19 ± 3 *^#^	>15	49 ± 4 *^#^	3.1 ± 0.9 *^#^
**12-oxo-Calanolide A**	18 ± 2 *^#^	>15	36 ± 8 *^#^	1.9 ± 0.1 *^#^
**Calanolide A**	14 ± 1 *	4.1 ± 0.3 *^#^	52 ± 8 *^#^	8.2 ± 1.7 *^#^
**Inocalophyllin B**	20 ± 2 *	> 15	41 ± 5 *^#^	4.2 ± 1.3 *^#^
**Oxacillin**	30 ± 1	ND^e^	ND	ND
**Streptomycin**	ND	0.4 ± 0.1	ND	ND

Results are expressed as means ± SEM of 3 independent experiments made in triplicate. ^a^ Antimicrobial activity was evaluated by measuring the diameter of the zone of inhibition impregnated at 20 µg/disk. ^b^ MIC is defined as the minimum inhibitory concentration. ^c^ IC_50_ is defined as the concentration of compound resulting in 50% viability after 48 h and calculated from concentration–response curves. ^d^ Fraction P contains ~70% in weight of inophyllum P. ^e^ ND, not determined. Statistics: * *p* < 0.05 vs. TO and ^#^ *p* < 0.05 vs. EtTO, by one-way ANOVA followed by Newman-Keuls test.

## Data Availability

Not applicable Please refer to suggested Data Availability Statements in section “MDPI Research Data Policies” at https://www.mdpi.com/ethics.

## Supplementary Materials

The following are available online at https://www.mdpi.com/2076-3921/10/2/199/s1, Figures S1–S18, ^1^H-NMR and ^13^C-NMR spectra, and MS analysis of isolated and identified phytoconstituents of Tamanu oil, and the structure of detected DEPMPO spin adducts.

## Abbreviations

AAPH, 2,2′-diazobis(2-amidinopropane)dihydrochloride; ATR, acidic fraction; DEPMPO, 5-(diethoxyphosphoryl)-5 methyl-1-pyrroline *N*-oxide; DMEM, Dulbecco’s modified eagle’s medium; DPPH, 1,1-diphenyl-2-picrylhydrazyl; EPR, electron paramagnetic resonance; EtTO, Tamanu oil ethanolic extract; LDH, lactate dehydrogenase; LPO, lipoxygenase; MDA, malondialdehyde; MeETR, Tamanu resin methanolic extract; MeTO, Tamanu oil methanolic extract; MTT, 3-(4,5-dimethylthiazol-2-yl)-2,5-diphenyltetrazolium bromide; NTR, neutral fraction; PBS, phosphate-buffered saline; PTA, proteinase K; ROS, reactive oxygen species; TBA, 2-thiobarbituric acid; TFC, total flavonoid content; TO, Tamanu oil; TPC, total phenolic content.
