# Supplementary material for: Improving the Antioxidant Properties of Calophyllum inophyllum Seed Oil from French Polynesia: Development and Biological Applications of Resinous Ethanol-Soluble Extracts
Source: Antioxidants (Basel). 2021 Jan 30;10(2):199. doi: 10.3390/antiox10020199 (PMC7910904; doi:10.3390/antiox10020199)

Supplementary Information

Improving the antioxidant properties of *Calophyllum inophyllum L.* seed oil from French Polynesia: development and biological applications of ethanol-soluble resins extracts

Mathieu Cassien [1,2,3], Anne Mercier [1], Sophie Thétiot-Laurent [1], Marcel Culcasi [1], Alice Asteian [1], Emilie Ricquebourg [1], Gaëtan Herbette [4], Jean-Pierre Bianchini [2], Phila Raharivelomanana [2] and Sylvia Pietri [1,*]

1- Aix Marseille Univ, CNRS, ICR, UMR7273, Marseille, France

2- Univ Polynésie Française, IFREMER, ILM, IRD, EIO UMR241, Tahiti, Polynésie Française

3- YELEN Analytics, Ensuès-la-Redonne, France

4- Aix Marseille Univ, CNRS, Spectropole, FSCM, Marseille, France

***Corresponding Author**

S. Pietri, PhD

Aix Marseille Univ, CNRS, ICR, UMR7273, Institut de Chimie Radicalaire
Sondes Moléculaires en Biologie et Stress Oxydant, Service 522
Faculté des Sciences de Saint-Jérôme, Avenue Escadrille Normandie-Niemen
13397 Marseille Cedex 20 - France
Tel: 33 (0)4 13 94 58 07 - Fax: 33 (0)4 91 28 87 58
E-Mail: sylvia.pietri@univ-amu.fr

Table of Contents

1. ^{1}H-NMR Spectra of Isolated or Identified Phytoconstituents of Tamanu Resins 2
Figure S1: ^{1}H-NMR spectrum of inocalophyllin B 3
Figure S2: ^{1}H-NMR spectrum of inophyllum C 3
Figure S3: ^{1}H-NMR spectrum of inophyllum E 4
Figure S4: ^{1}H-NMR spectrum of inophyllum P 4
Figure S5: ^{1}H-NMR spectrum of calophyllolide 5
Figure S6: ^{1}H-NMR spectrum of 12-oxocalanolide A 5
Figure S7: ^{1}H-NMR spectrum of calanolide A 6
Figure S8: ^{1}H-NMR spectrum of calanolide D 6
Figure S9: ^{1}H-NMR spectrum of calanolide GUT 70 7
Figure S10: ^{1}H-NMR spectrum of tamanolide 7
Figure S11: ^{1}H-NMR spectrum of tamanolide D 8
Figure S12: ^{1}H-NMR spectrum of Fraction D 8
Figure S13: ^{1}H-NMR spectrum of Fraction P 9

2. ^{13}C-NMR Spectra of some Isolated Phytoconstituents of Tamanu Resins 9
Figure S14: ^{13}C DEPT 135-NMR spectrum of inocalophyllin B 9
Figure S15: ^{13}C DEPT 135-NMR spectrum of calanolide A 10
Figure S16: ^{13}C DEPT 135-NMR spectrum of calanolide GUT 70 10
Figure S17. ^{13}C DEPT 135-NMR spectrum of tamanolide D 11

3. MS Analysis of Isolated Phytoconstituents of Tamanu Resins 11

4. EPR Spin Trapping 12
Scheme S1. Formation of HO• and O_2•$^-$/HOO• DEPMPO spin adducts 12

1. ^{1}H-NMR Spectra of Isolated or Identified Phytoconstituents of Tamanu Resins

Figure S1. ^{1}H-NMR spectrum of inocalophyllin B

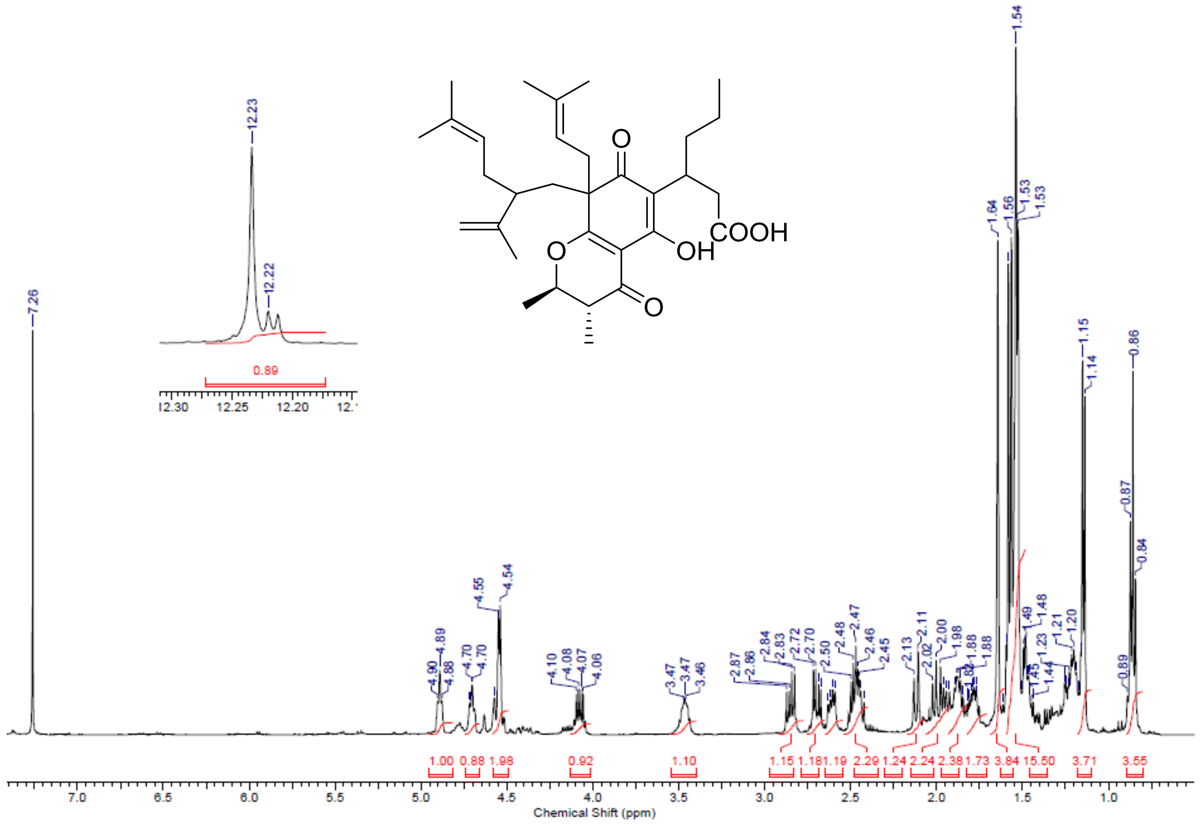

Figure S2. ^{1}H-NMR spectrum of inophyllum C

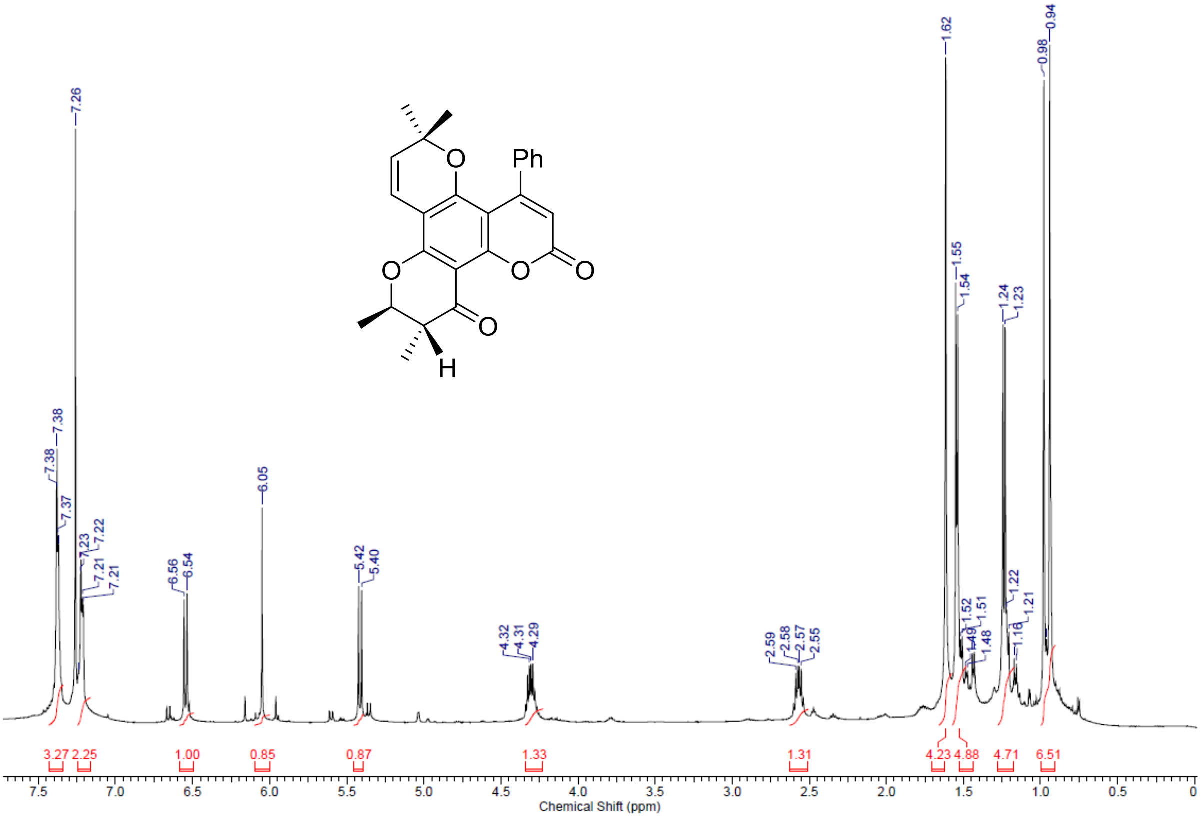

Figure S3. ^{1}H-NMR spectrum of inophyllum E

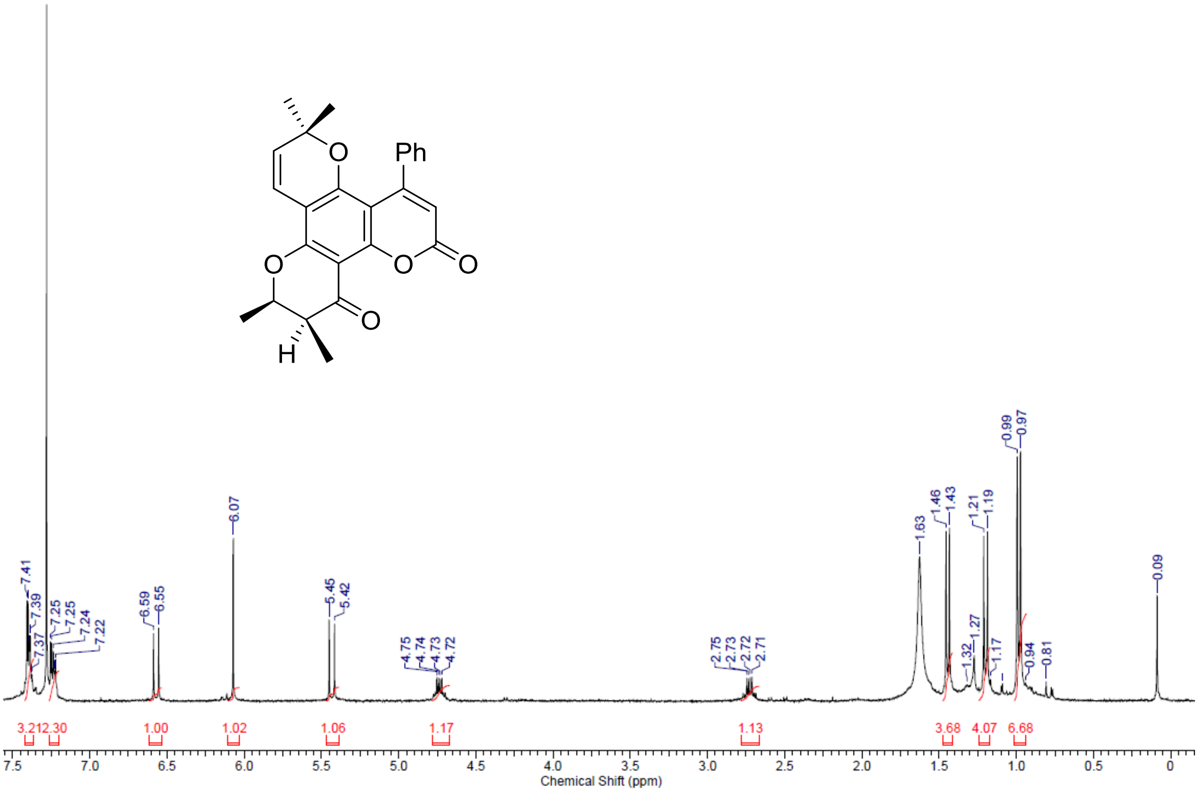

Figure S4. ^{1}H-NMR spectrum of inophyllum P

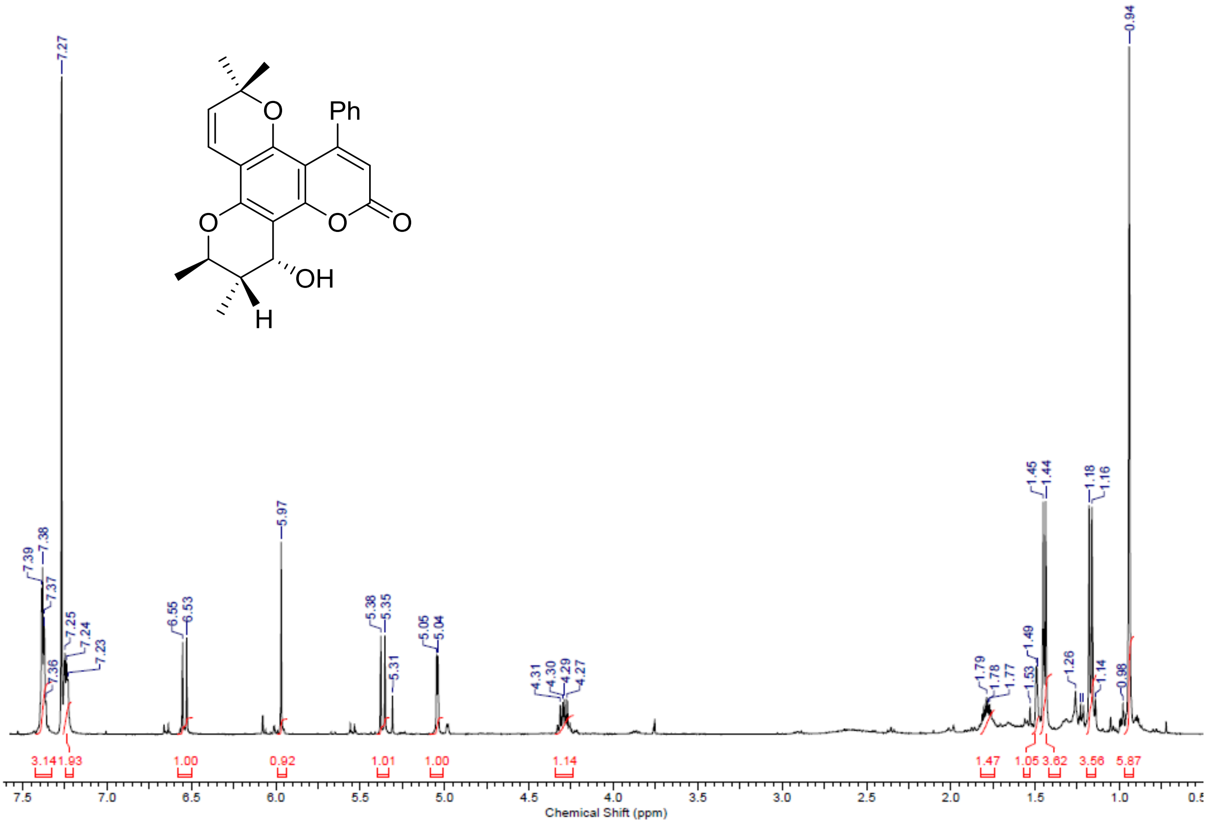

Figure S5. ^{1}H-NMR spectrum of calophyllolide

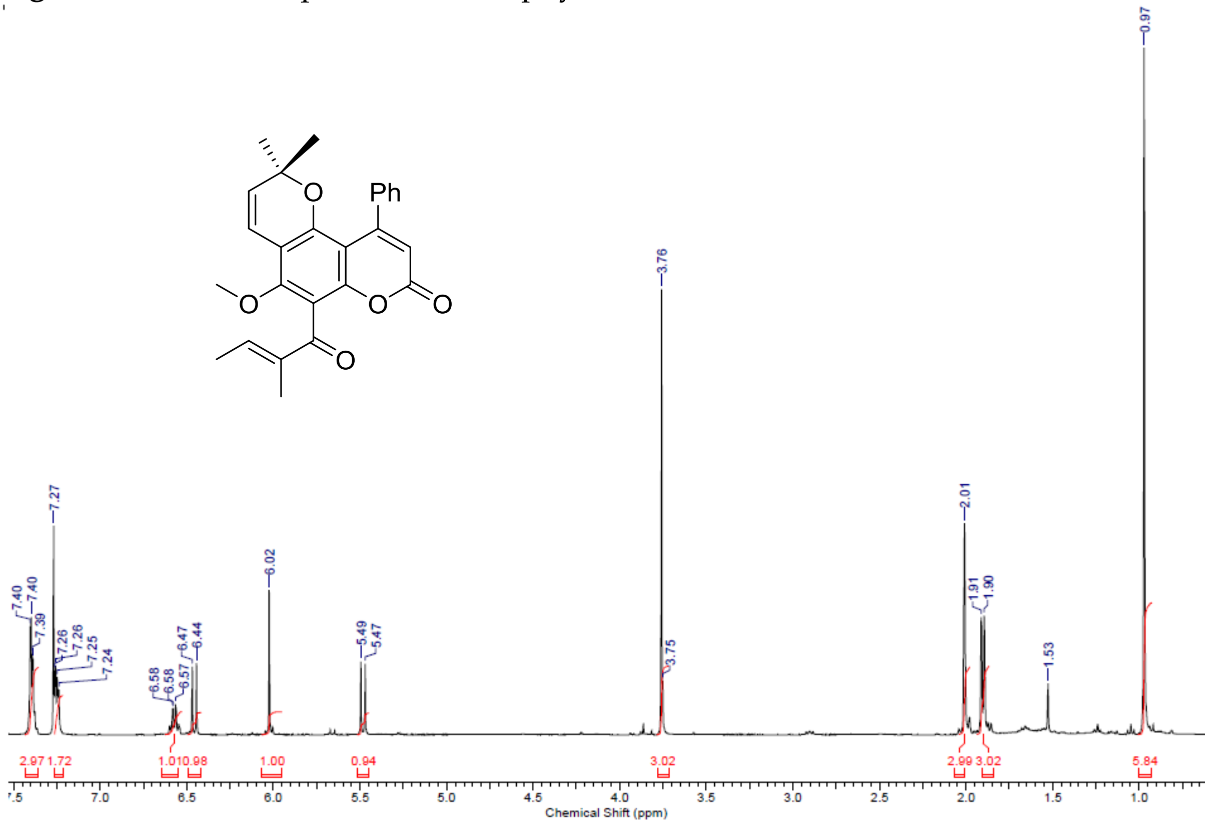

Figure S6. ^{1}H-NMR spectrum of 12-oxocalanolide A

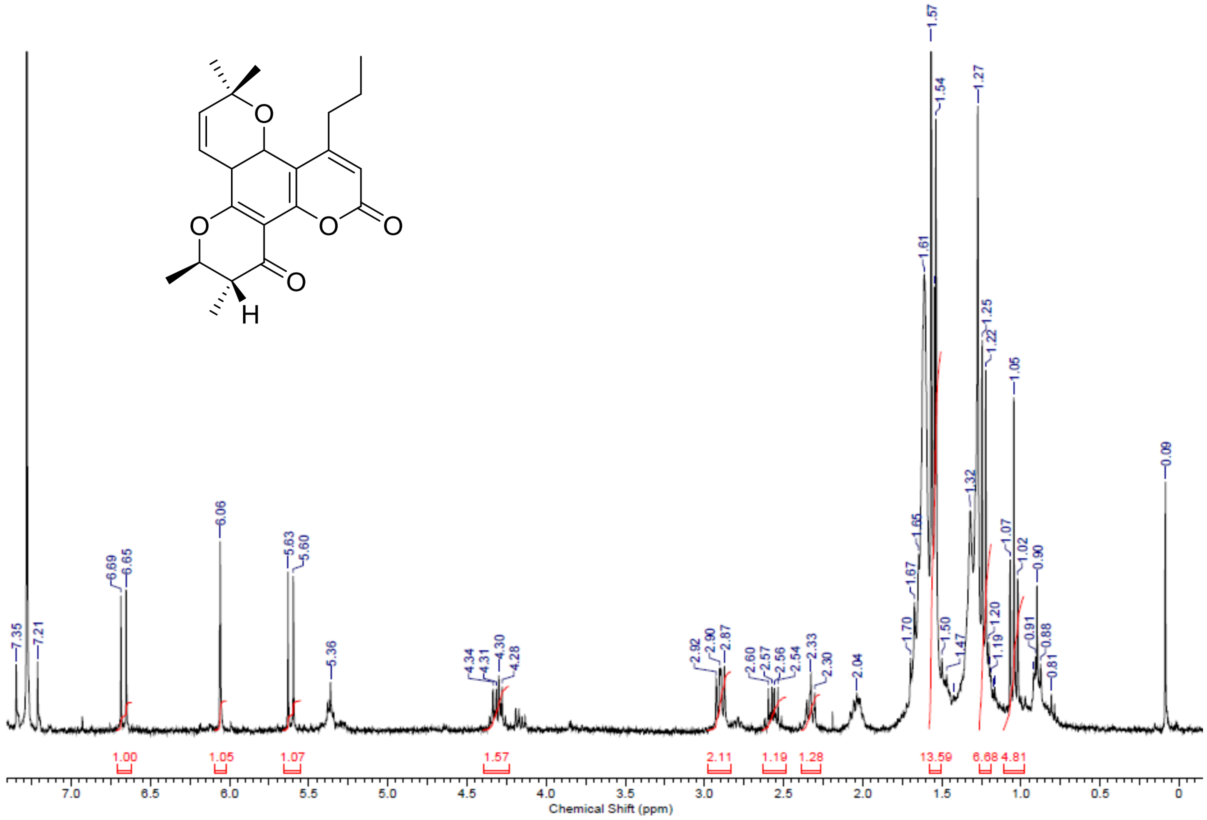

Figure S7. ^{1}H-NMR spectrum of calanolide A

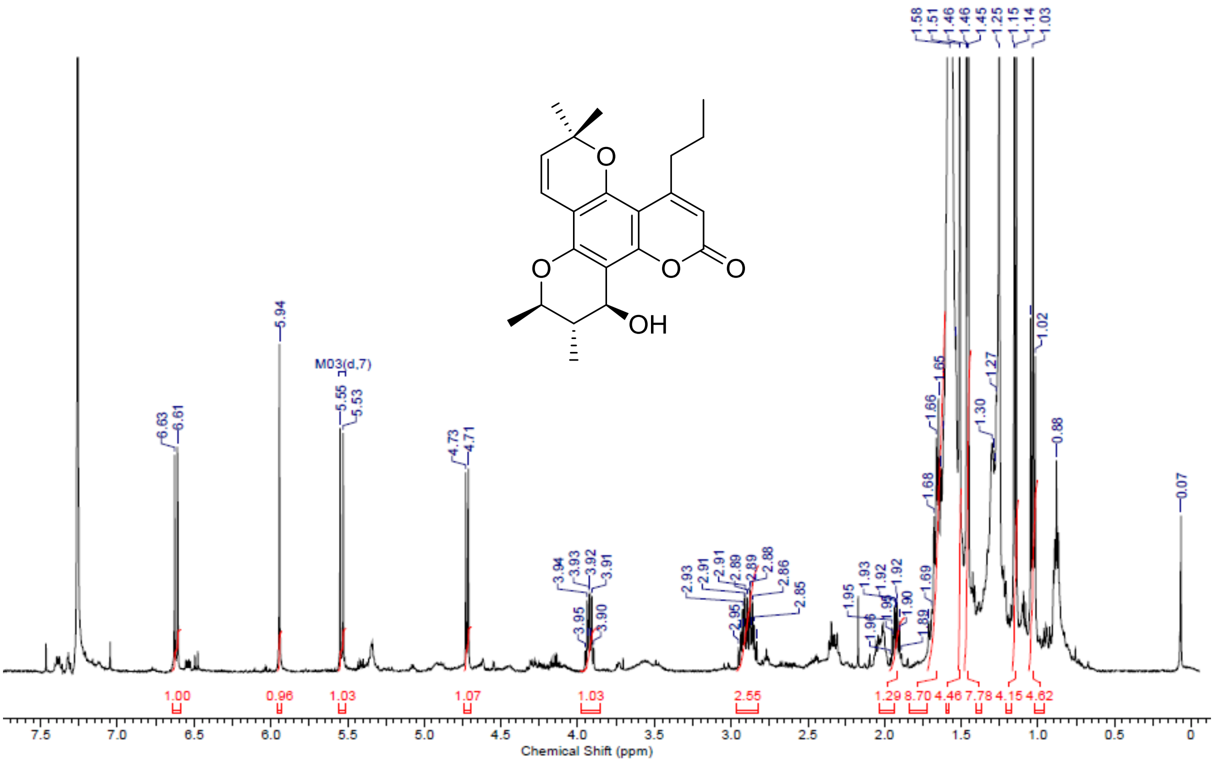

Figure S8. ^{1}H-NMR spectrum of calanolide D

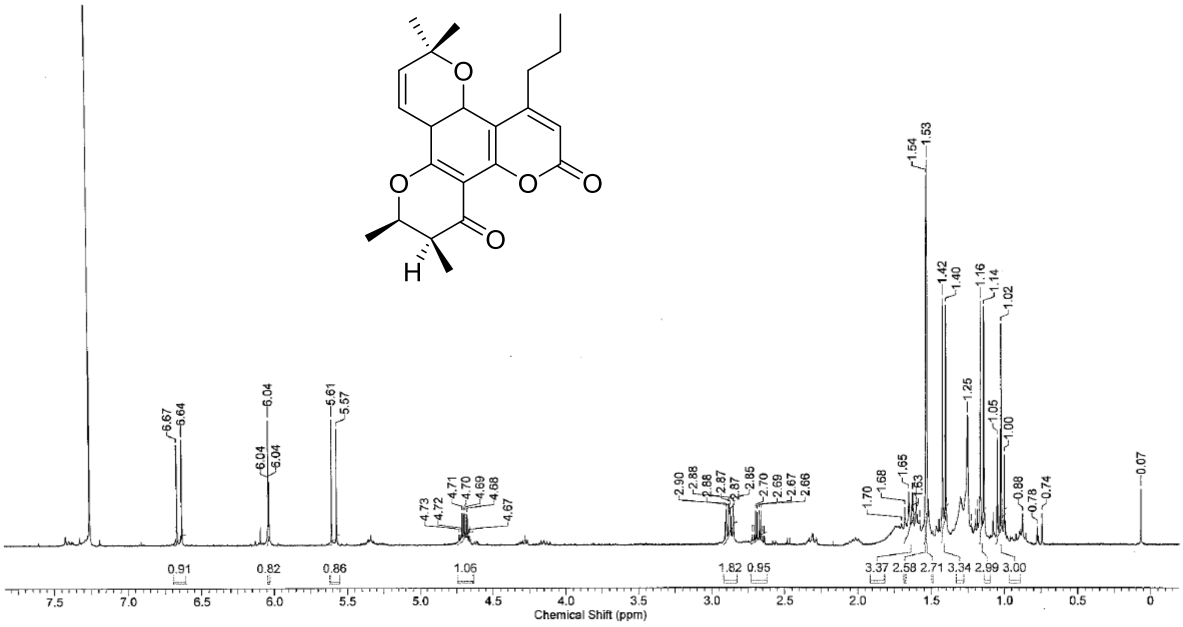

Figure S9. ^{1}H-NMR spectrum of calanolide GUT 70

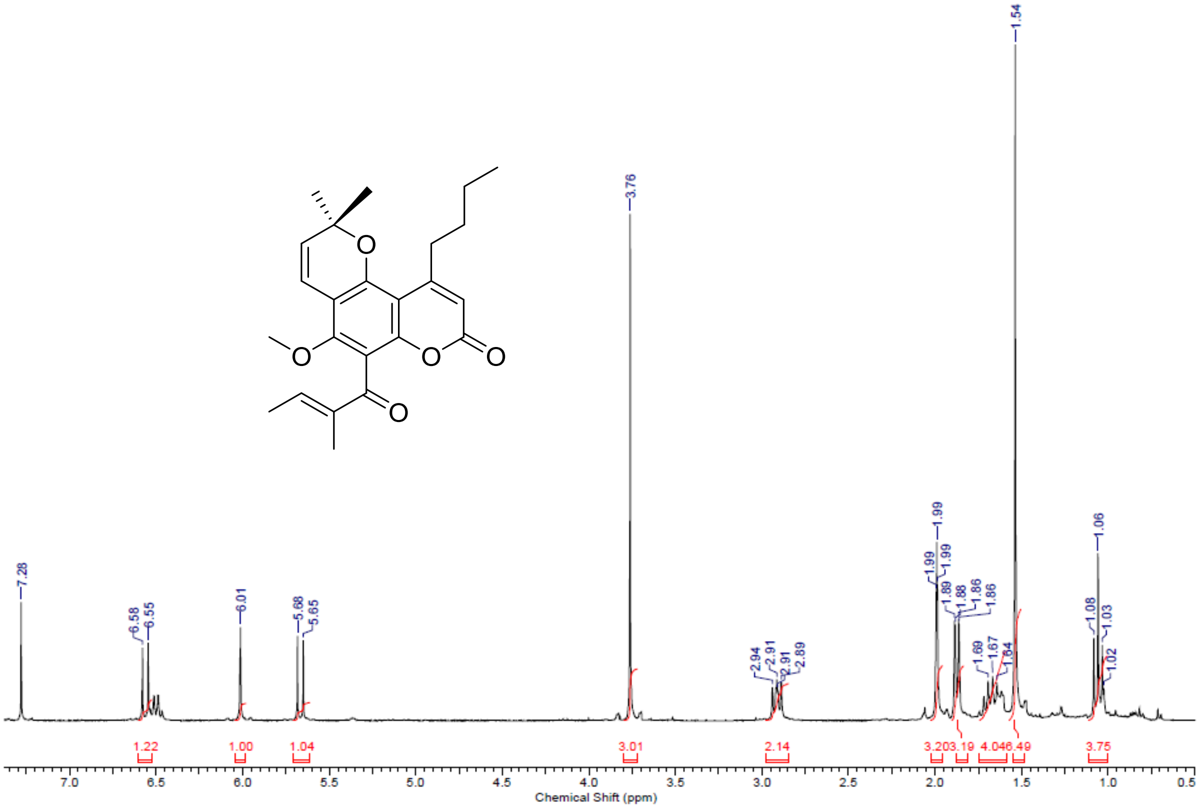

Figure S10. ^{1}H-NMR spectrum of tamanolide

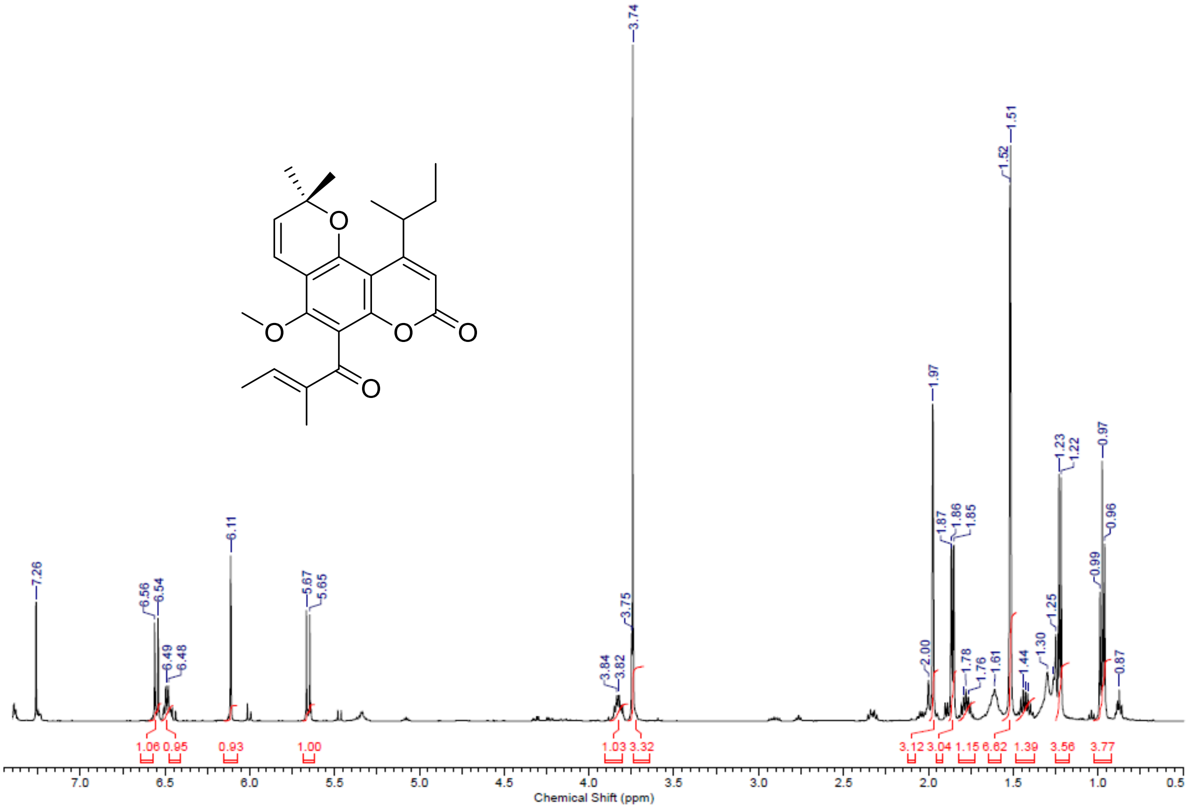

Figure S11. ^{1}H-NMR spectrum of tamanolide D

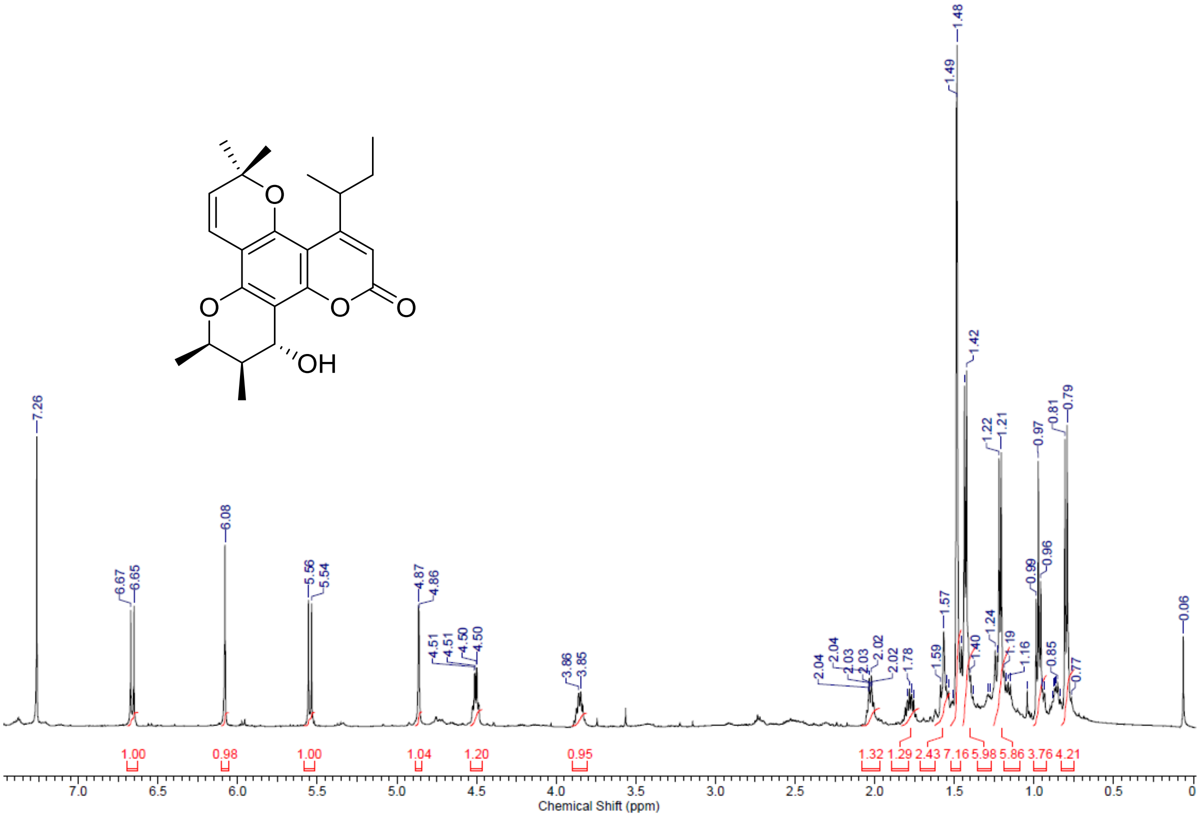

Figure S12. ^{1}H-NMR spectrum of Fraction D

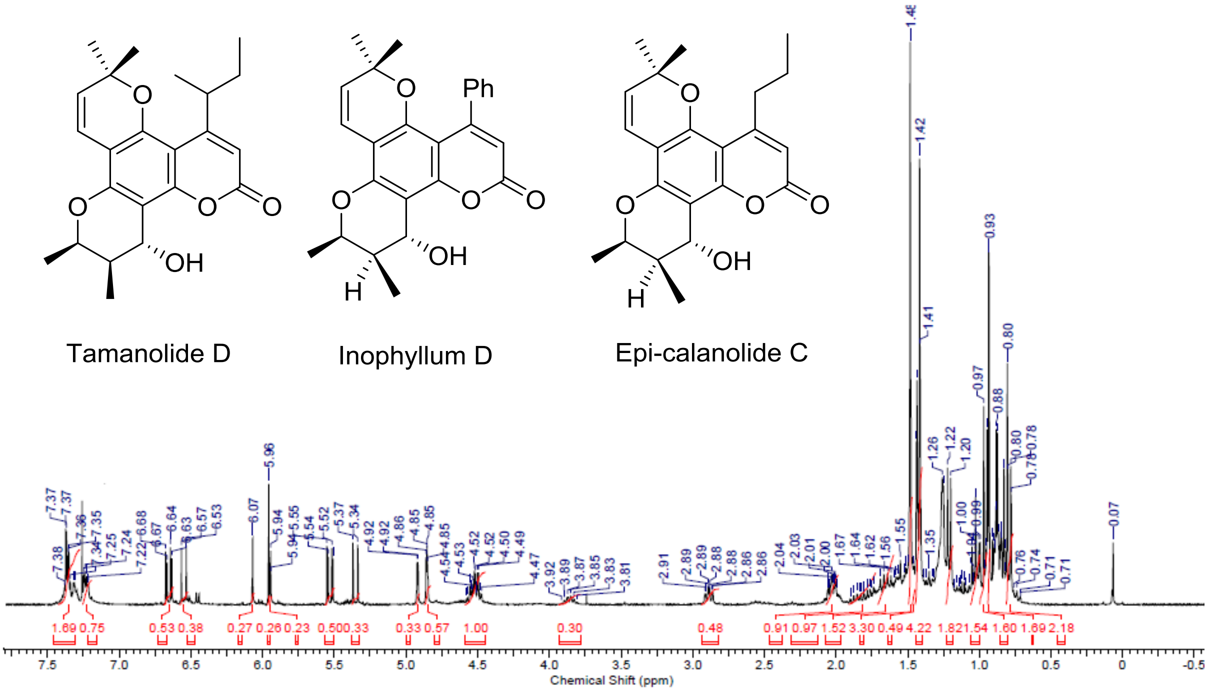

Figure S13. ^{1}H-NMR spectrum of Fraction P

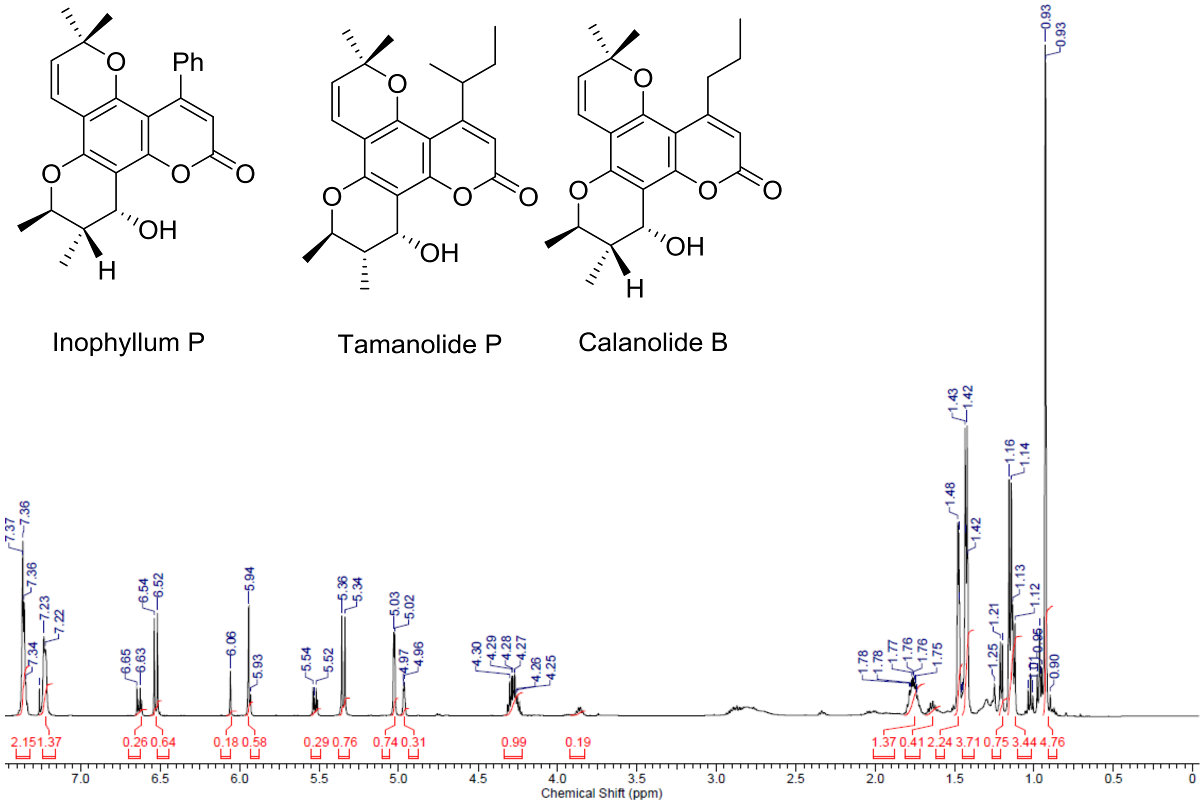

2. ^{13}C-NMR Spectra of some Isolated Phytoconstituents of Tamanu Resins

Figure S14. ^{13}C DEPT 135-NMR spectrum of inocalophyllin B

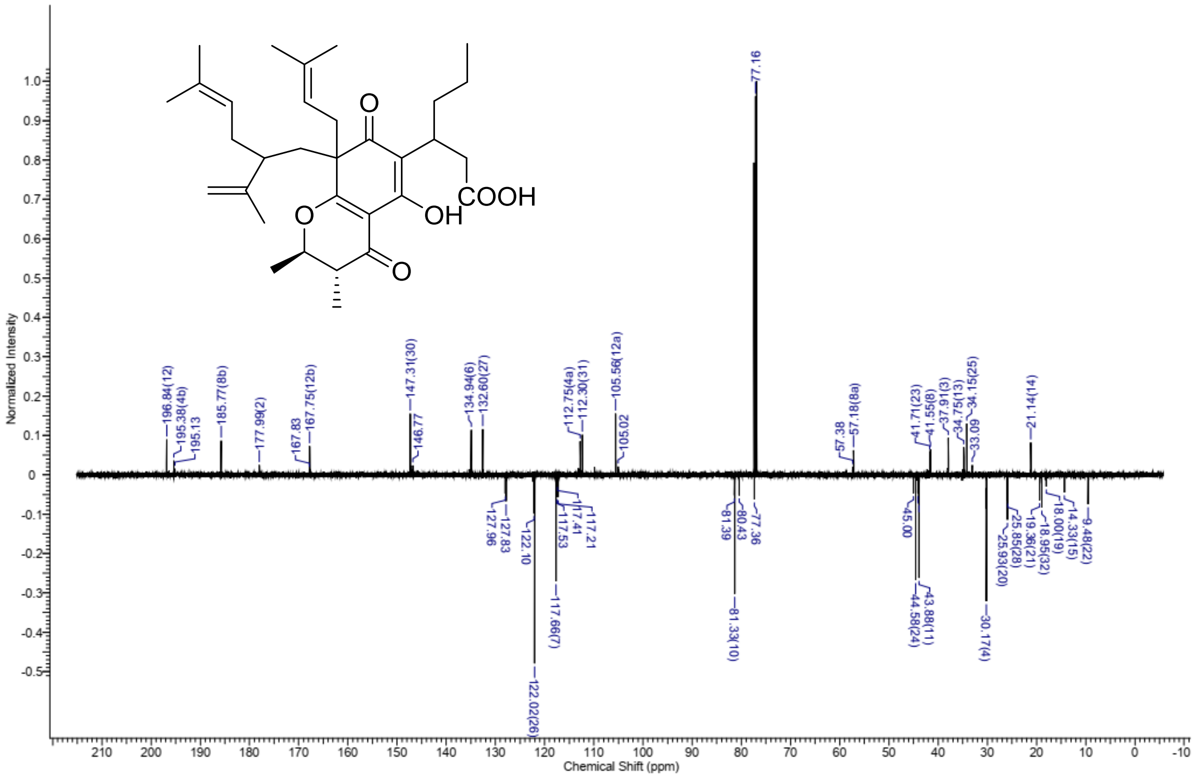

Figure S15: ^{13}C DEPT 135-NMR spectrum of calanolide A

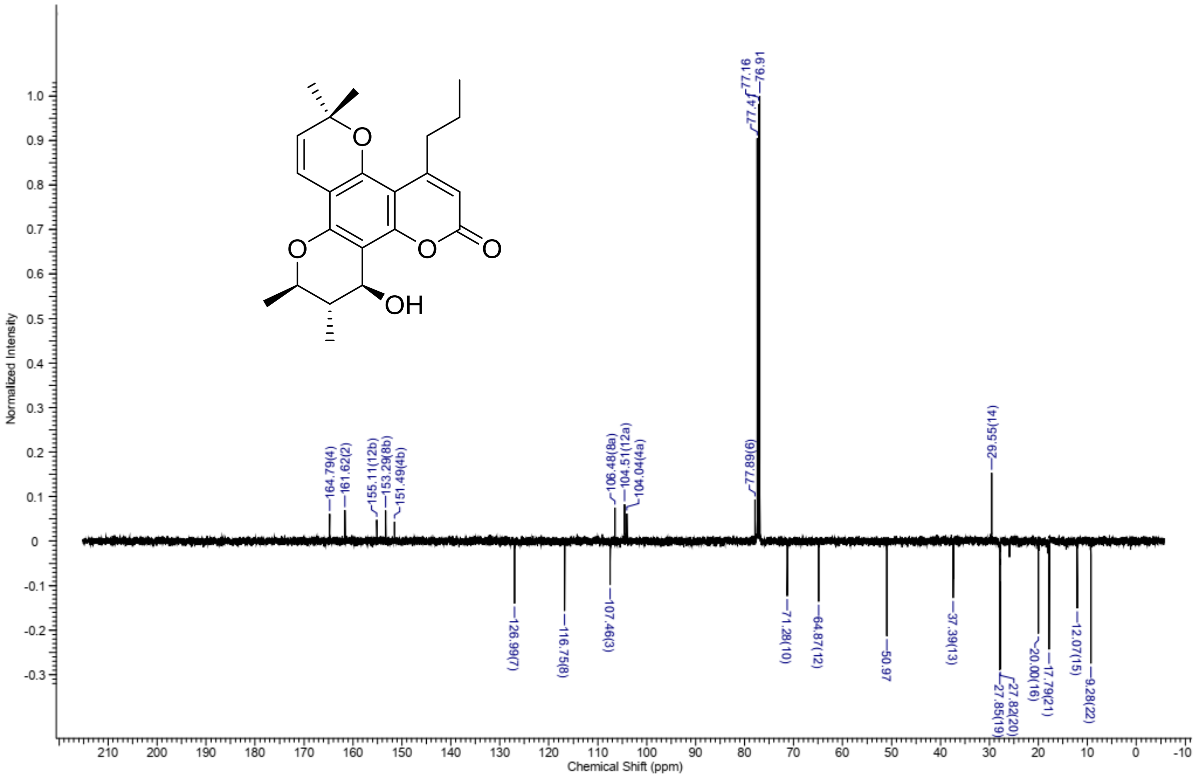

Figure S16: ^{13}C DEPT 135-NMR spectrum of calanolide GUT 70

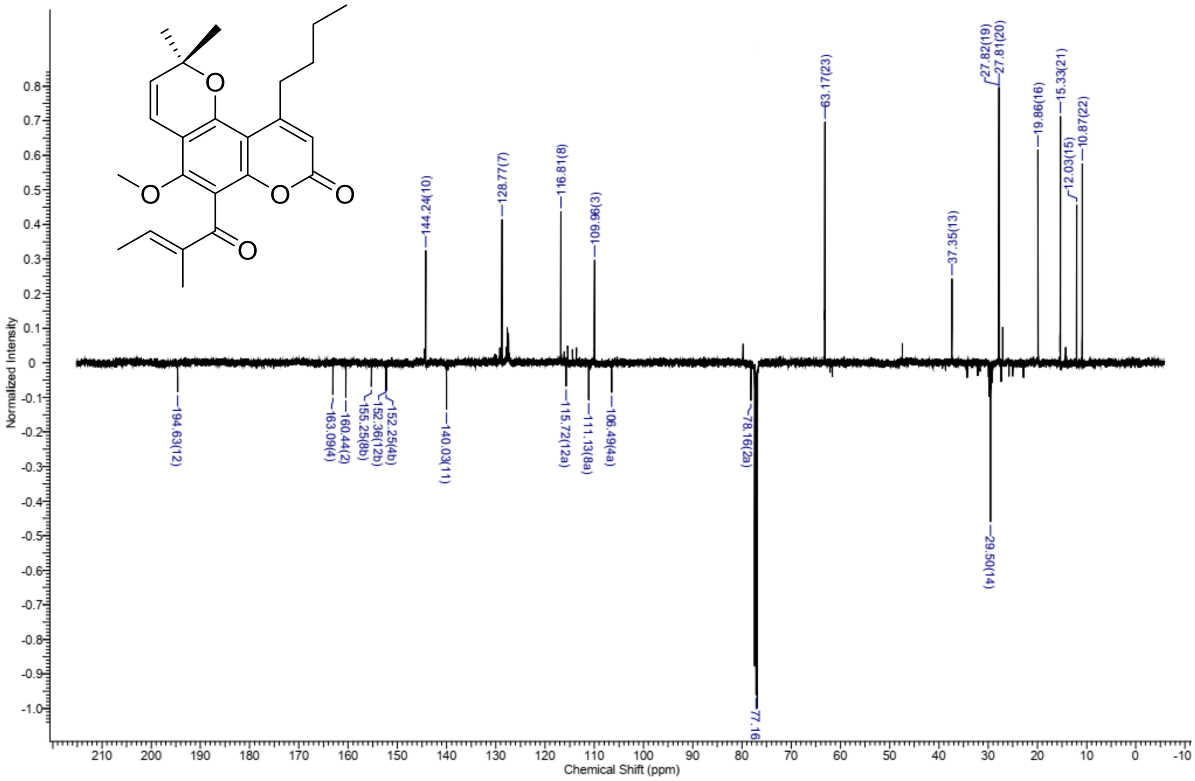

Figure S17: ^{13}C DEPT 135-NMR spectrum of tamanolide D

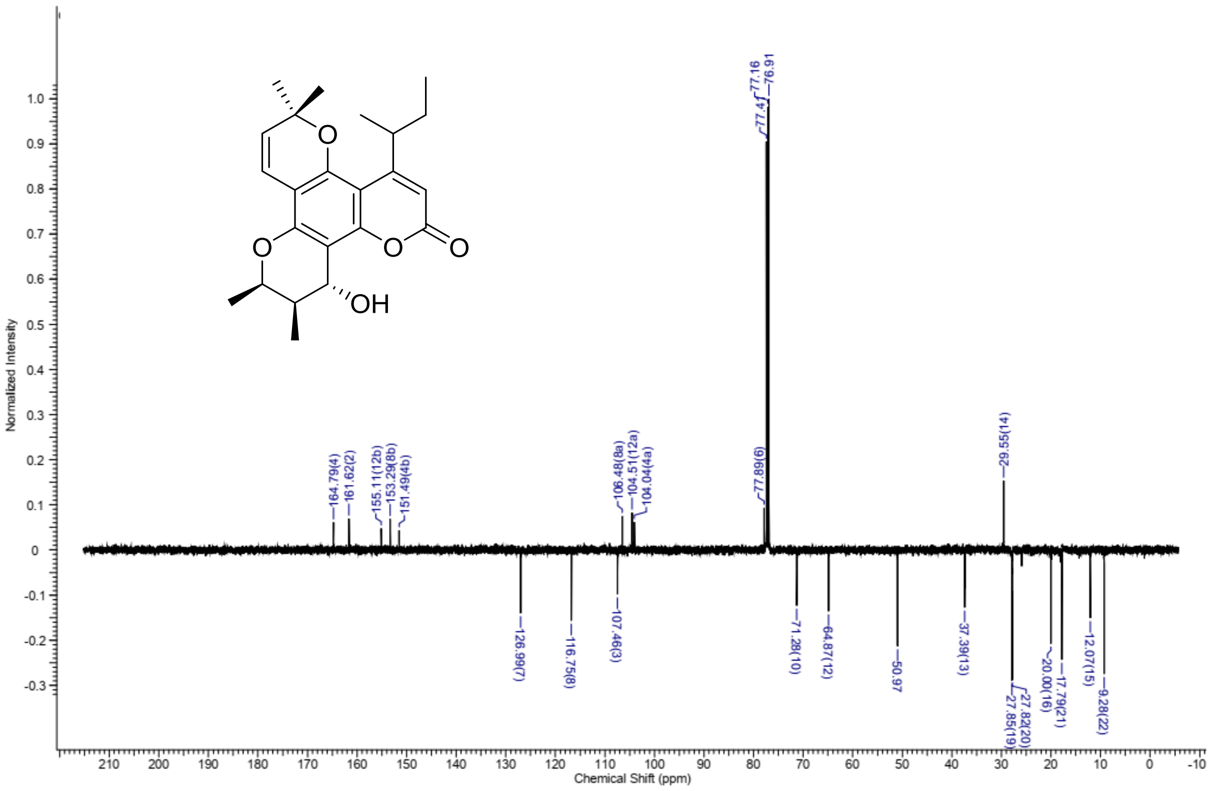

3. MS Analysis of Isolated Phytoconstituents of Tamanu Resins

The electron spray ionization mass spectra (ESI-MS) were recorded with a 3200 QTRAP (Applied Biosystems SCIEX) mass spectrometer in a positive ion mode to produce $[M + H]^+$ ions (Spectropole, Aix Marseille Université, Marseille, France).

Inocalophyllin B: ESI-MS (*m*/*z*) 527.34 corresponding to $[C_{32}H_{46}O_6 + H]^+$

Inophyllum C: ESI-MS (*m*/*z*) 403.15 corresponding to $[C_{25}H_{22}O_5 + H]^+$

Inophyllum E: ESI-MS (*m*/*z*) 403.15 corresponding to $[C_{25}H_{22}O_5 + H]^+$

Inophyllum P: ESI-MS (*m*/*z*) 405.17 corresponding to $[C_{25}H_{24}O_5 + H]^+$

Calophyllolide: ESI-MS (*m*/*z*) 417.17 corresponding to $[C_{26}H_{24}O_5 + H]^+$

12-oxocalanolide A: ESI-MS (*m*/*z*) 369.17 corresponding to $[C_{22}H_{24}O_5 + H]^+$

Calanolide A: ESI-MS (*m*/*z*) 371.19 corresponding to $[C_{22}H_{26}O_5 + H]^+$

Calanolide D: ESI-MS (*m*/*z*) 369.17 corresponding to $[C_{22}H_{24}O_5 + H]^+$

Calanolide GUT 70: ESI-MS (*m*/*z*) 383.19 corresponding to $[C_{23}H_{26}O_5 + H]^+$

Tamanolide: ESI-MS (*m*/*z*) 397.19 corresponding to $[C_{24}H_{28}O_5 + H]^+$

Tamanolide D: ESI-MS (*m*/*z*) 385.20 corresponding to $[C_{23}H_{28}O_5 + H]^+$

4. EPR Spin Trapping

Scheme S1. Formation of HO• and ($O_2^{\bullet-}$/HOO•) DEPMPO spin adducts

The main EPR coupling nuclei of the detected spin adducts are displayed in red. Stereochemistry is shown for the major *trans*-DEPMPO-OOH adduct.

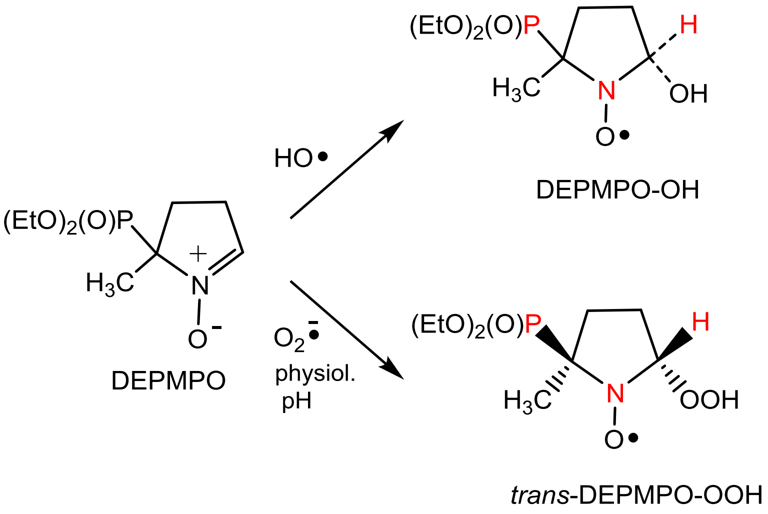

Supplement: Supplementary file 1 [file antioxidants-10-00199-s001.pdf]
